# Novel protective and risk loci in hip dysplasia in German Shepherds

**DOI:** 10.1371/journal.pgen.1008197

**Published:** 2019-07-19

**Authors:** Lea I. Mikkola, Saila Holopainen, Anu K. Lappalainen, Tiina Pessa-Morikawa, Thomas J. P. Augustine, Meharji Arumilli, Marjo K. Hytönen, Osmo Hakosalo, Hannes Lohi, Antti Iivanainen

**Affiliations:** 1 Department of Veterinary Biosciences, University of Helsinki, Helsinki, Finland; 2 Department of Molecular Genetics, Folkhälsan Institute of Genetics, Helsinki, Finland; 3 Research Programs Unit, Molecular Neurology, University of Helsinki, Helsinki, Finland; 4 Department of Equine and Small Animal Medicine, University of Helsinki, Helsinki, Finland; Stanford University School of Medicine, UNITED STATES

## Abstract

Canine hip dysplasia is a common, non-congenital, complex and hereditary disorder. It can inflict severe pain via secondary osteoarthritis and lead to euthanasia. An analogous disorder exists in humans. The genetic background of hip dysplasia in both species has remained ambiguous despite rigorous studies. We aimed to investigate the genetic causes of this disorder in one of the high-risk breeds, the German Shepherd. We performed genetic analyses with carefully phenotyped case-control cohorts comprising 525 German Shepherds. In our genome-wide association studies we identified four suggestive loci on chromosomes 1 and 9. Targeted resequencing of the two loci on chromosome 9 from 24 affected and 24 control German Shepherds revealed deletions of variable sizes in a putative enhancer element of the *NOG* gene. *NOG* encodes for noggin, a well-described bone morphogenetic protein inhibitor affecting multiple developmental processes, including joint development. The deletion was associated with the healthy controls and mildly dysplastic dogs suggesting a protective role against canine hip dysplasia. Two enhancer variants displayed a decreased activity in a dual luciferase reporter assay. Our study identifies novel loci and candidate genes for canine hip dysplasia, with potential regulatory variants in the *NOG* gene. Further research is warranted to elucidate how the identified variants affect the expression of noggin in canine hips, and what the potential effects of the other identified loci are.

## Author summary

Hip dysplasia is a common orthopedic disorder in dogs and humans. It can pose a serious welfare problem with severe pain. The genetic background of this disorder remains inconclusive even after years of arduous research. We used the genotypes of 525 German Shepherds with carefully determined hip scores to identify genomic regions potentially harboring genetic risk factors for the disorder. We found four regions on chromosomes 1 and 9 exhibiting suggestive association with the disorder phenotypes. Further analysis of the identified loci on chromosome 9 by sequencing 48 dogs revealed deletions in a potential regulatory region of *NOG* - the gene encoding noggin, a known regulator of joint development in mice and in humans. Using a reporter assay, we demonstrated that the deletions decrease the enhancer activity of the regulatory region and could therefore affect the expression of *NOG* in hips. The deletions significantly differentiate the healthy and the mild phenotypes from the moderate-to-severe phenotypes. Therefore, our results suggest that the deletion protects against hip dysplasia. Future research should focus on how these regulatory variants affect the expression of noggin in canine hips, and what the roles of noggin and the other revealed loci are in canine hip dysplasia.

## Introduction

Many hereditary disorders appear in both humans and dogs with gene variants from common ancestral genes affecting the disease [1]. Canine hip dysplasia (CHD) is a non-congenital disease, causing skeletal abnormalities in growing dogs, the first signs appearing at the age of three to four months [2]. CHD is defined as the laxity of the joint, resulting in instability and subluxation [2,3]. The femoral head is not completely covered by the acetabulum, which then leads to increased force over smaller surface area due to incongruence of the coxofemoral joint [2,3]. This in turn causes microfractures in the acetabulum and the femoral head [2,3], detrition of the articular cartilage, inflammation of the synovial membrane and secondary osteoarthritis (OA) [3]. CHD can be painful up to a point where it poses a serious welfare problem. Hip dysplasia appears in humans in divergent forms from infancy to adolescence and to adulthood [4]. Of these, the adolescent hip dysplasia is clinically and developmentally closest to CHD [5].

Official scoring of CHD varies by country: in Finland it is scored categorically from A (normal) to E (severely affected hip joints with possible OA) using ventrodorsal extension radiographs as standardized by the Finnish Kennel Club under the Fédération Cynologique Internationale (FCI) [6]. The scoring is based on the following features: level of congruence between the femoral head and the acetabulum, degree of subluxation of the joint, Norberg angle, shape of the femoral head and neck, shape and depth of the acetabulum, and signs of secondary OA [6,7]. Other traits also add into hip morphology, but they are not routinely evaluated [8]. In the end, only the categorical score for each hip joint is recorded and made available for later use.

The severity of CHD depends on environmental and genetic factors [2,3,9–23]. CHD prevalence varies also by breeds and breed groups [5], but is common for example in German Shepherds with a reported 37% prevalence in Finland between 2000–2017 (6413/16433) [24]. The heritability (h^2^) estimates for the hip score are generally moderate across breeds (0.20–0.38) [12–14], although in German Shepherds the h^2^ estimates have varied from 0.1 to 0.6 as summarized in [18]. In a Finnish German Shepherd population the estimates were 0.31–0.35 [25]. In earlier studies the h^2^ estimates for the traits determining the hip score (e.g. Norberg angle, secondary OA, articular congruence and dorsolateral subluxation) have varied considerably from low h^2^ = 0.10 to high h^2^ = 0.73 [10,11,14,15,19]. Some evidence of major genes affecting CHD exist from studies utilizing variance estimates and Bayesian modeling [16,18,26,27]. Also, a recent study investigating quantivative trait locus (QTL) associations with CHD revealed a large effect locus on chromosome 28, which had a 6° additive effect on Norberg angle values in Golden Retrievers and Labrador Retrievers [28]. Fels and Distl suggested QTLs on chromosomes 19, 24, 26 and 34, which associated with CHD in German Shepherds [29]. In addition, a number of other small effect loci and potential candidate genes have been found [15,21,23]. The reported QTLs and candidate genes are inconsistent between studies, however, as both Sánchez-Molano et al. [15] and Zhu et al. [22] have discussed. Ultimately, different study populations and methods affect the results substantially, which must be recognized when reviewing the data. *FBN2* encoding for fibrillin 2 is to our knowledge the only gene in which a mutation has been demonstrated to be associated with CHD using gene expression analysis [30]. However, Lavrijsen et al. [23] found no evidence of association between the region harboring *FBN2* and CHD, but they suggested this discrepancy may be caused by differences in Dutch and U.S. Labrador Retriever populations.

We have carried out genome-wide association studies (GWAS) in case-control cohorts, revealing a total of four associated loci on two chromosomes. Subsequent sequencing of the underlying region on one chromosome identified putative regulatory variants of *NOG*, which downregulated a reporter gene expression *in vitro*, and were associated with the healthy and the mildly dysplastic phenotypes.

## Results

### Novel CHD loci on chromosomes 1 and 9

**Fig 1:**
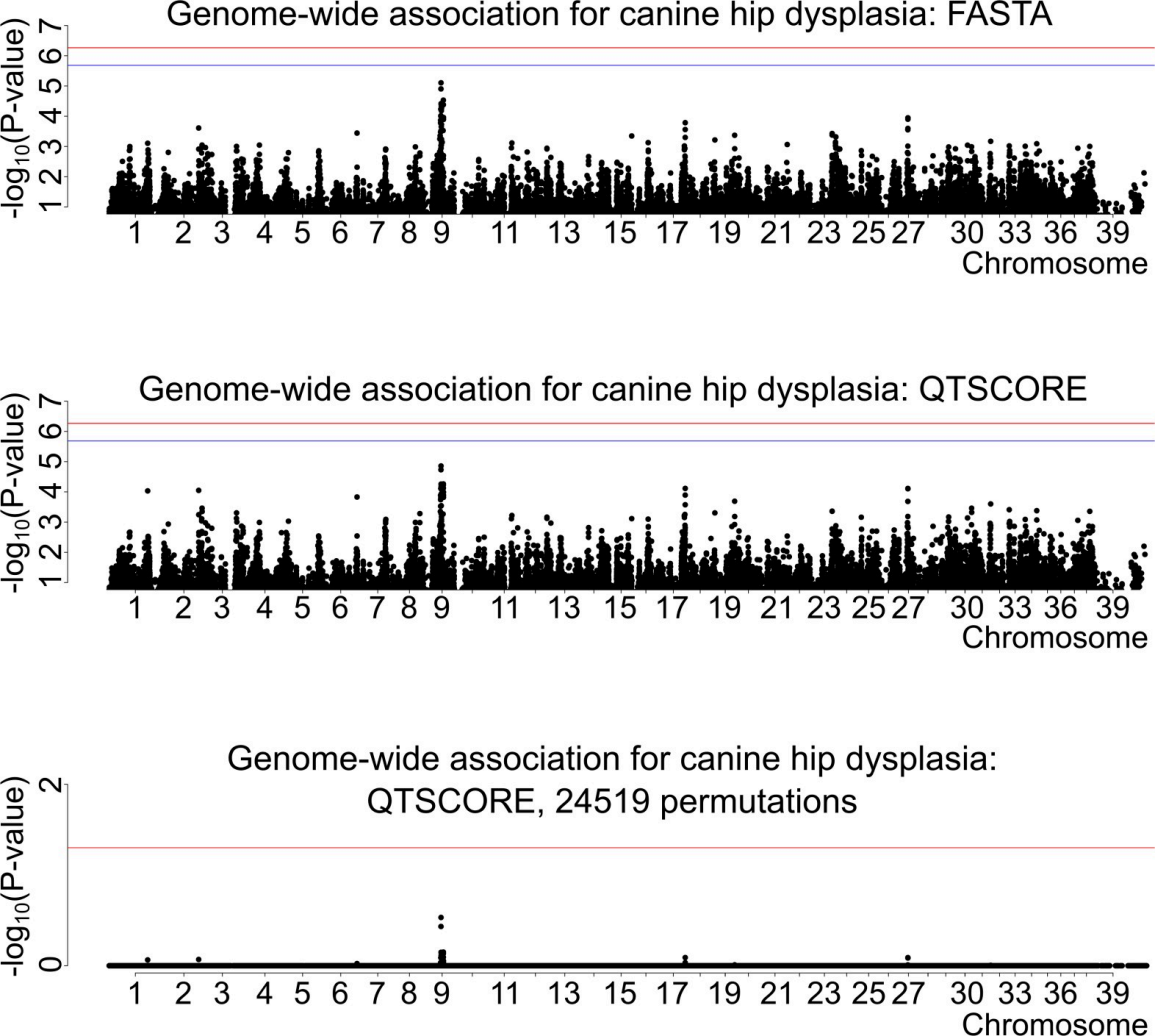
Genome-wide association for canine hip dysplasia: FASTA and QTSCORE, N(cases) = 132, N(controls) = 160. For the upper two figure segments the red horizontal lines are the thresholds for Bonferroni correction for significance. The blue horizontal line is the threshold for significance for independent tests. In the undermost segment, where the permuted P-values are shown, the single red line represents the threshold for genome-wide significance level of 0.05.

**Fig 2:**
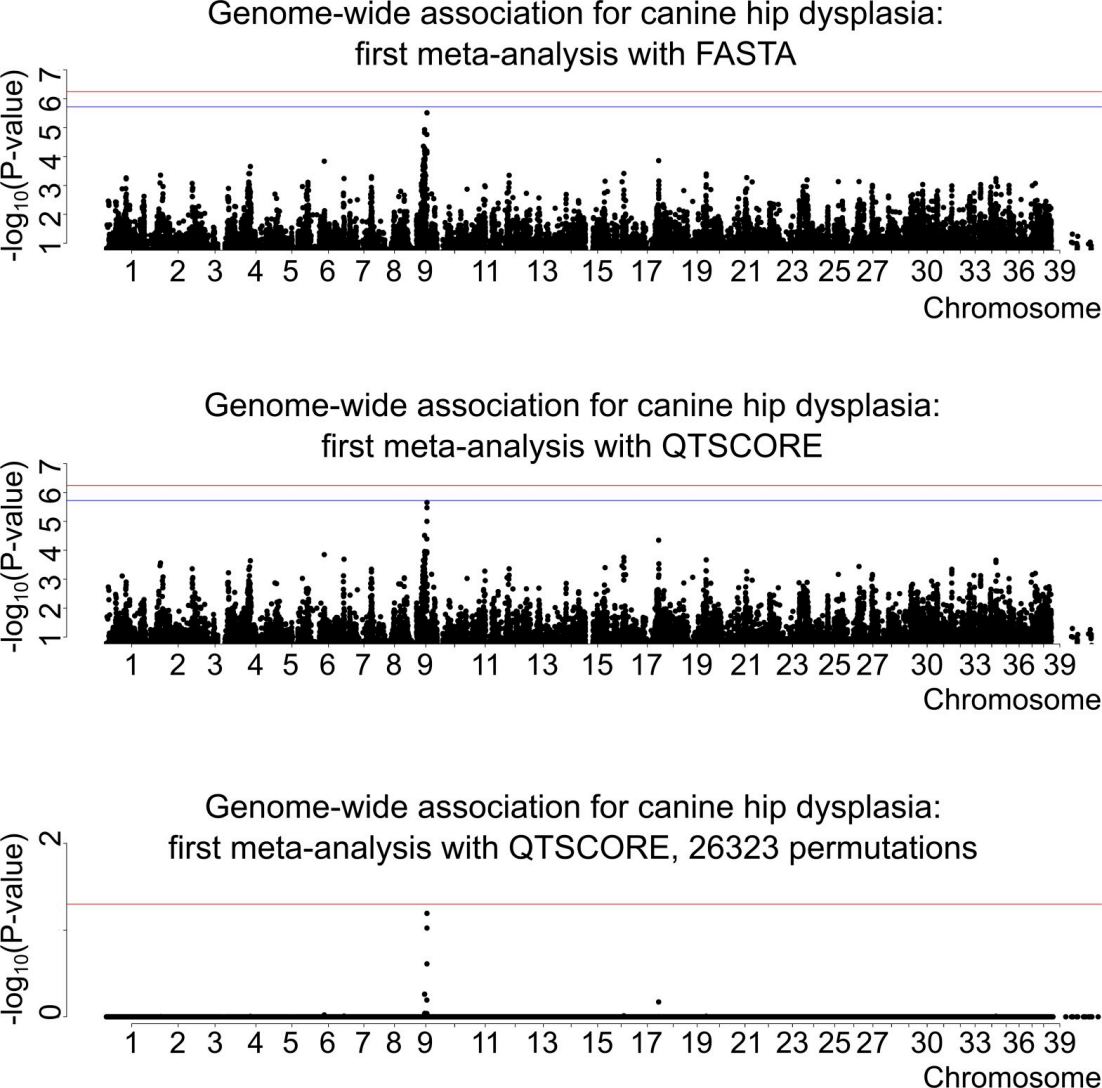
Genome-wide association for canine hip dysplasia: First meta-analysis, N(cases) = 132, N(controls) = 277. For the upper two figure segments the red horizontal line is the threshold for Bonferroni correction for significance. The blue horizontal line is the threshold for significance for independent tests. In the undermost segment, where the permuted P-values are shown, the single red line represents the threshold for genome-wide significance level of 0.05.

**Fig 3:**
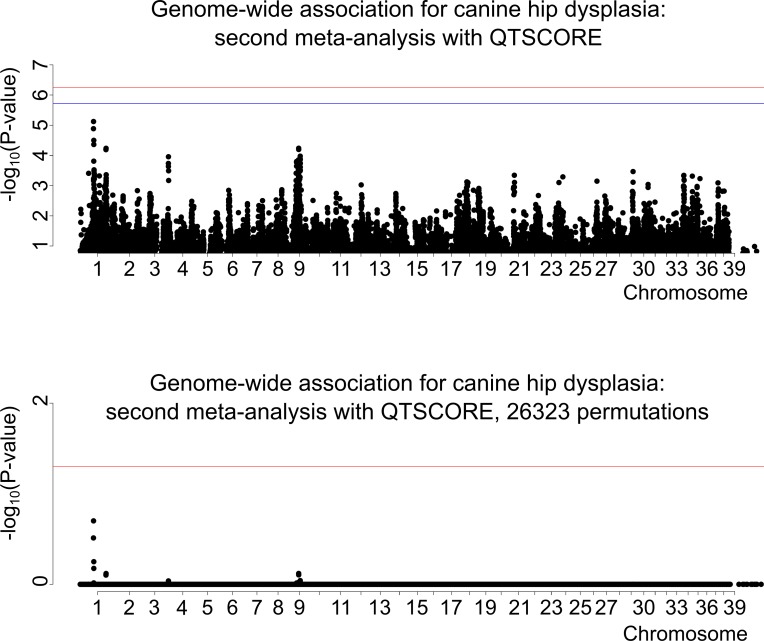
Genome-wide association for canine hip dysplasia: Second meta-analysis, N(cases) = 247, N(controls) = 277. For the upper figure segment the red horizontal line is the threshold for Bonferroni correction for significance. The blue horizontal line is the threshold for significance for independent tests. In the undermost segment, where the permuted P-values are shown, the single red line represents the threshold for genome-wide significance level of 0.05.

To map CHD loci, we originally performed GWAS using the Illumina 173K chip with 160 controls (with A/A hip scores, left/right hip) and 132 cases (D/D, D/E, E/D or E/E), which revealed a suggestive association on canine chromosome 9 (Fig 1). Subsequently, we genotyped 233 more individuals and analyzed the data from all 525 dogs. However, one control was dropped from the subsequent meta-analyses due to a missing genotyping batch covariate. Therefore, the first meta-analysis cohort comprised 277 controls and 132 cases (with D/D, D/E, E/D or E/E hip scores) (Fig 2), and the second, less stringent meta-analysis included the same controls and 247 cases (with C/C, C/D, D/C, D/D, D/E, E/D or E/E hip scores; dogs with C/C are later on referred as mild cases) (Fig 3). None of the top SNPs from the different analyses reached genome-wide significance. The data were analyzed using two different statistical methods within the R package "GenABEL": FASTA and QTSCORE. In the original association analysis and the first meta-analysis QTSCORE was used with environmental residual to be comparable with FASTA. In the second meta-analysis QTSCORE was used with standard genomic control [31]. We decided to use FASTA because it is effective in handling highly stratified data of related individuals, while it usually is not as conservative as QTSCORE. However, for the second meta-analysis we used only QTSCORE with standard genomic control, because FASTA ended up losing more power (S1 Fig). QTSCORE was used to obtain the permuted P-values for genome-wide significance in all analyses, because FASTA cannot be used for this task. Inflation factor lambda, describing possible inflation of test statistics due to population stratification, was estimated as 1.02 for FASTA, deflation of 0.76 for QTSCORE for the original analysis and 1.01 and 0.72 for the first meta-analysis respectively. For the second meta-analysis lambda was 1.43. When lambda was < 1, the deflation was corrected with reverse genomic control [32]. The P-values after inflation and deflation factor corrections, after permutation tests with QTSCORE, and genotypic and allelic odds ratios (OR) with the respective 95% confidence intervals (CI) for all of the top SNPs are shown in Table 1 (original GWAS), Table 2 (first meta-analysis), and Table 3 (second meta-analysis). The association on chromosome 9 in the original genome-wide association analysis was 14 times stronger, and in the first meta-analysis over 45 times stronger than in any other loci in the genome (Fig 1 and Fig 2). In the second meta-analysis the association on chromosome 1 was over seven times stronger than what was observed on chromosome 9, and over 14 times stronger than for any other loci in the genome (Fig 3).

**Table 1:** Adjusted P-values, empirical genome-wide significance levels and odds ratios for the most significant SNPs from the original genome-wide association analyses.

SNP name (CanFam3.1)	Alleles (Risk / Non-risk)	Locus	GWAS (N = 292)				
			FASTA: P-value after inflation factor correction (lambda = 1.02)	QTSCORE: P-value after inflation factor correction (lambda = 0.76)	Genome-wide significance (24159 permutations, lambda = 0.76)	Genotypic odds ratios with 95% CI (OR_Aa_; OR_AA_)	Allelic odds ratios (OR_A_)
TIGRP2P126044	C/T	Chr9: 30993502	5.8x10-5	7.7x10-5	0.82	0.32 [0.17–0.61]; 0.18 [0.09–0.36]	0.41 [0.29–0.57]
BICF2S23027935	A/G	Chr9: 31300189	1.2x10-5	1.9x10-5	0.36	0.29 [0.15–0.55]; 0.17 [0.08–0.34]	0.40 [0.29–0.56]
BICF2P742007	C/T*	Chr9: 31387114	7.8x10-6	1.4x10-5	0.29	0.61 [0.34–1.09]; 0.18 [0.09–0.35]	0.38 [0.27–0.53]
BICF2G630834826	A/G	Chr9: 31477907	3.8x10-5	5.5x10-5	0.71	0.60 [0.34–1.06]; 0.22 [0.11–0.42]	0.43 [0.31–0.60]
BICF2G630835002	C/T	Chr9: 31772306	4.1x10-5	6.6x10-5	0.77	0.61 [0.34–1.09]; 0.20 [0.10–0.39]	0.42 [0.30–0.59]
BICF2G630835188	G/T	Chr9: 32166146	4.6x10-5	8.1x10-5	0.83	0.42 [0.23–0.77]; 0.24 [0.13–0.46]	0.45 [0.32–0.63]
BICF2G630835223	G/A	Chr9: 32271830	5.2x10-5	7.2x10-5	0.79	0.57 [0.32–1.01]; 0.22 [0.11–0.42]	0.43 [0.31–0.60]
BICF2P283506	T/C	Chr9: 32382532	5.5x10-5	8.0x10-5	0.83	0.56 [0.32–0.99]; 0.23 [0.12–0.44]	0.44 [0.32–0.61]
BICF2G630837209	G/A	Chr9: 36543581	4.2x10-5	5.9x10-5	0.73	2.55 [1.32–4.94]; 4.90 [2.44–9.85]	2.28 [1.63–3.19]
BICF2G630837233	G/T	Chr9: 36565413	4.2x10-5	7.5x10-5	0.80	2.55 [1.32–4.94]; 4.77 [2.38–9.57]	2.25 [1.61–3.15]
BICF2G630837240	A/G	Chr9: 36579921	2.9x10-5	5.5x10-5	0.71	2.32 [1.19–4.52]; 4.58 [2.29–9.15]	2.32 [1.60–3.16]

OR_Aa_, odds ratio for genotype Aa relative to the genotype aa; OR_AA_, odds ratio for genotype AA relative to the genotype aa; OR_A_, odds ratio for allele A relative to the allele a. Only the SNPs that showed association with the disorder after both statistical methods (FASTA and QTSCORE) with P-value threshold of 1.0x10-4 were included.

*Complementary strand to what is indicated in Table 2.

**Table 2:** Adjusted P-values, empirical genome-wide significance levels and odds ratios for the top SNPs after the meta-analysis in moderate-to-severe cases compared with controls.

SNP name (CanFam3.1)	Alleles (Risk / Non-risk)	Locus	GWAS meta-analysis (N = 409)				
			FASTA: P-value after inflation factor correction (lambda = 1.01)	QTSCORE: P-value after inflation factor correction (lambda = 0.72)	Genome-wide significance (26323 permutations, lambda = 0.72)	Genotypic odds ratios with 95% CI (OR_Aa_; OR_AA_)	Allelic odds ratios (OR_A_)
BICF2S23027935	A/G	Chr9: 31300189	1.5x10-5	3.1x10-5	0.55	0.59 [0.36–0.96]; 0.19 [0.10–0.36]	0.43 [0.32–0.58]
BICF2P742007	G/A*	Chr9: 31387114	1.2x10-5	3.1x10-5	0.54	0.49 [0.30–0.81]; 0.17 [0.09–0.31]	0.39 [0.29–0.53]
BICF2G630837240	A/G	Chr9: 36579921	1.7x10-5	4.1x10-5	0.64	2.00 [1.07–3.73]; 4.78 [2.51–9.09]	2.28 [1.68–3.10]
BICF2G630837405	A/G	Chr9: 36837067	3.0x10-6	3.4x10-6	0.090	0.37 [0.22–0.61]; 0.21 [0.12–0.38]	0.44 [0.33–0.59]
BICF2P272135	G/C	Chr9: 36842872	7.6x10-5	1.0x10-5	0.24	0.36 [0.22–0.60]; 0.20 [0.11–0.37]	0.43 [0.32–0.58]
TIGRP2P126345	G/A	Chr9: 36886621	3.1x10-6	2.2x10-6	0.060	0.35 [0.21–0.58]; 0.22 [0.12–0.40]	0.43 [0.32–0.58]

*The strand of SNP BICF2P742007 was flipped in the pre-meta-analysis data merge and therefore the alleles are complementary to those in Table 1. All the SNPs in the table showed association with the disorder after both statistical methods (FASTA and QTSCORE) at the P-value threshold of 1.0x10-4.

**Table 3:** Adjusted P-values, empirical genome-wide significance levels and odds ratios for the top SNPs after the meta-analysis of mild to severe cases compared with controls.

SNP name (CanFam3.1)	Alleles (Risk / Non-risk)	Locus	GWAS meta-analysis (N = 524)			
			QTSCORE: P-value after inflation factor correction (lambda = 1.43)	Genome-wide significance (87158 permutations, lambda = 1.43)	Genotypic odds ratios with 95% CI (OR_Aa_; OR_AA_)	Allelic odds ratios (OR_A_)
BICF2S23248027	G/C	Chr1: 45161186	1.3x10-5	0.31	0.62 [0.37–1.04]; 0.27 [0.16–0.47]	0.52 [0.41–0.68]
BICF2P468585	C/A	Chr1: 45382633	7.5x10-6	0.20	0.61 [0.36–1.02]; 0.26 [0.16–0.47]	0.51 [0.40–0.65]
BICF2P1037296	A/C	Chr1: 46268586	4.4x10-5	0.67	0.56 [0.35–0.90]; 0.29 [0.17–0.49]	0.54 [0.42–0.69]
BICF2P357728	G/A	Chr1: 46279297	3.2x10-5	0.56	0.55 [0.34–0.89]; 0.28 [0.17–0.47]	0.53 [0.41–0.68]
BICF2S23329752	G/A	Chr1: 87382164	6.4x10-5	0.79	0.32 [0.15–0.66]; 0.21 [0.10–0.43]	0.52 [0.40–0.68]
BICF2S23660342	G/A	Chr1: 87446951	6.2x10-5	0.78	0.32 [0.15–0.68]; 0.20 [0.09–0.42]	0.52 [0.40–0.68]
BICF2P1129598	C/A	Chr1: 87749401	5.8x10-5	0.76	0.34 [0.15–0.76]; 0.20 [0.09–0.44]	0.51 [0.38–0.68]
BICF2S23027935	A/G	Chr9: 31300189	5.8x10-5	0.76	0.74 [0.48–1.11]; 0.30 [0.18–0.49]	0.54 [0.41–0.68]
BICF2P742007	G/A	Chr9: 31387114	6.4x10-5	0.79	0.67 [0.42–1.02]; 0.32 [0.19–0.50]	0.53 [0.41–0.68]

Only the SNPs that showed association with the disorder at least at the P-value threshold of 1.0x10-4 were included.

The SNPs on chromosome 9 represent two separate loci; there was moderate to high linkage disequilibrium (LD) between the markers within each locus (r^2^ = 0.64–0.99), but limited LD between the loci (r^2^ = 0.34–0.56) (S1 Table). Clumping analysis, a tool to estimate the number of independently associated loci, corroborated this by revealing two loci within the targeted region on chromosome 9 (S2 Fig). OR for the SNPs within the first locus (Chr9: 30993502–32382532) were all less than one (Table 1), and for the markers within the second locus (Chr9: 36543581–36579921) OR varied between 2.25 and 4.90 (Table 1).

The results from the first meta-analysis were similar to the original GWAS (Table 2). We observed a 2.6x difference in the smallest P-values, indicating a small gain in power, when we compared the SNPs with the strongest association in both analyses: 7.8x10-6 in the original GWAS (BICF2P742007) and 3.0x10-6 in the first meta-analysis (BICF2G630837405). The LD-structure of the top SNPs from this analysis (S1 Table) also resembled the respective values seen in the original GWAS, indicating two independent loci. The odds ratios implied a protective role for the top SNPs within the first locus as in the original GWAS. In the second locus, BICF2G630837240 locating at Chr9:36579921 had OR of 2.00–4.78 (Table 2), which again was comparable to the values observed in the original GWAS. The other SNPs in this second locus, locating within 36837067–36886621, were only observed in this meta-analysis, although they exhibited the strongest association to the disorder (Table 2). These three SNPs were in moderate LD (0.68–0.71) with BICF2G630837240, but had odds ratios < 1 (Table 2).

As in the original GWAS and the first meta-analysis with the stringent phenotype definition, some of the top SNPs demonstrated notable LD between each other (r2 = 0.80–1.00; S1 Table) in the second meta-analysis using the relaxed phenotype. The clumping procedure indicated two loci on chromosome 1 and two loci on chromosome 9 (S3 Fig). The first associated locus on chromosome 1 spans over ~1.1 Mb region, and all of the ORs imply a protective association (OR = 0.26–0.62; Table 3). The second locus is ~367 kb long and ORs for all the three SNPs are also < 1 (Table 3). The two loci on chromosome 9 corresponded to the same regions that have been defined before. However, the association of the second locus on chromosome 9 reached a P-value of 1.1x10-4 at best (BICF2G630837209) and was therefore not included in Table 3.

Table 4 summarizes the four loci and their representative SNPs that displayed the strongest association with the disorder over the analyses. All of the top SNPs in the ~1.1 Mb locus on chromosome 1 were in high LD with each other (S1 Table). One of the associated SNPs is intronic to NADPH Oxidase 3 (*NOX3*) (BICF2S23248027), and the rest of the SNPs lie in an intergenic region between *NOX3* and AT-Rich Interaction Domain 1B (*ARID1B*) (BICF2P468585, BICF2P1037296, and BICF2P357728) (Table 4). The SNPs closest to *ARID1B* were BICF2P357728 and BICF2P1037296, located 91234 bp and 101945 bp upstream. The second locus on chromosome 1 included many genes (Table 4). The corresponding SNPs were also in high LD with each other (r^2^ = 0.84–0.96, S1 Table) and were located either within MAM Domain Containing 2 (*MAMDC2*) (BICF2S23329752 and BICF2S23660342) or within Protein Prenyltransferase Alpha Subunit Repeat Containing 1 (*PTAR1*) (BICF2P1129598).

**Table 4:** Protein coding genes near the top SNPs from the genome-wide association analyses.

SNP ID (CanFam 3.1)	Location	Protein coding genes within ± 500 kb from the SNP	Relation to the closest gene(s)
BICF2S23248027	Chr1: 45161186	*ENSCAFG00000000562*, *SCAF8*, *TIAM2*, *TFB1M*, *CLDN20*, ***NOX3***	Intronic: *NOX3*
BICF2P468585	Chr1: 45382633	SCAF8, *TIAM2*, *TFB1M*, *CLDN20*, ***NOX3***, *ARID1B*	Intergenic: *NOX3* and *ARID1B*
BICF2P1037296	Chr1: 46268586	***ARID1B***, *TMEM242*, *ZDHHC14*	Intergenic: *NOX3* and *ARID1B*
BICF2P357728	Chr1: 46279297	***ARID1B***, *TMEM242*, *ZDHHC14*	Intergenic: *NOX3* and *ARID1B*
BICF2S23329752	Chr1: 87382164	*TRPM3*, *KLF9*, *SMC5*, ***MAMDC2***, *C9orf135*, *PTAR1*, *APBA1*	Intronic: *MAMDC2*
BICF2S23660342	Chr1: 87446951	*TRPM3*, *KLF9*, *SMC5*, ***MAMDC2***, *C9orf135*, *PTAR1*, *APBA1*	Intronic: *MAMDC2*
BICF2P1129598	Chr1: 87749401	*SMC5*, *MAMDC2*, *C9orf135*, ***PTAR1***, *APBA1*, *FAM189A2*, *TJP2*, *ENSCAFG00000013130*	Intronic: *PTAR1*
BICF2S23027935	Chr9: 31300189	*PCTP*, *ENSCAFG00000024313*, ***ANKFN1***, *NOG*, *C17orf67*, *DGKE*, *TRIM25*, *COIL*, *SCPEP1*	Intronic: *ANKFN1*
BICF2P742007	Chr9: 31387114	*ENSCAFG00000024313*, ***ANKFN1***, ***NOG***, *C17orf67*, *DGKE*, *TRIM25*, *COIL*, *SCPEP1*, *AKAP1*	Intergenic: *ANKFN1* (33207 bp downstream) and *NOG* (66839 bp upstream)
BICF2G630837240	Chr9: 36579921	*C17orf64*, *ENSCAFG00000017793*, *ENSCAFG00000023331*, *CA4*, *PSMG4*, *ZNHIT3*, *MYO19*, *PIGW*, *GGNBP2*, *DHRS11*, ***MRM1***, ***LHX1***, *AATF*, *ACACA*	Intergenic: *MRM1* (101495 bp downstream) and *LHX1* (178408 bp upstream)

Genes closest to the SNPs are in bold.

The top SNPs on chromosome 9 span over a region of ~ 5.3 Mb. BICF2S23027935 locates to an intron of Ankyrin-Repeat and Fibronectin Type III Domain Containing 1 (*ANKFN1*) and 153764 bp upstream from *NOG*, a known bone morphogenetic protein (BMP) inhibitor (Table 4). BICF2P742007 is intergenic and lies close to *NOG* (66839 bp upstream). The SNP representing the second locus on chromosome 9 (BICF2G630837240) is situated between the genes for Mitochondrial rRNA Methyltransferase 1 (*MRM1*) and LIM Homeobox 1 (*LHX1*) (Table 4).

### Mild cases resemble controls for the chromosome 9 loci but are more similar to moderate-to-severe cases for the locus on chromosome 1

We compared the genotype frequencies of the top markers (Table 5) between the different phenotype groups to assess if any of the loci behave differently in these comparisons. Here we did the following comparisons: controls (hip scores A/A) to mild cases (hip scores C/C) and mild cases to moderate-to-severe cases (hip scores C/D, D/C or worse), because other comparisons were covered in the in the meta-analyses described above. The allele and genotype frequencies of healthy dogs and mildly dysplastic dogs did not differ significantly on either chromosome (Table 5). Interestingly, the allele and genotype frequencies between mild cases and moderate-to-severe cases were significantly different on chromosome 9, but not on chromosome 1(Table 5). Odds ratios for all the significant comparisons indicated a protective association for the locus near *NOG* on chromosome 9 (Table 5: BICF2S23027935 and BICF2P742007). The second locus on chromosome 9, near *LHX1*, increased the odds for hip dysplasia about 2- to 5-fold in all the significant comparisons (Table 5: BICF2G630837240).

**Table 5:** Allelic and genotypic odds ratios between phenotype comparisons for the top markers on chromosomes 1 and 9.

			Comparison groups							
			Controls (N = 278); mild cases (N = 79)				Mild cases (N = 79); moderate-to-severe cases (N = 168)			
Chr	SNP	Allele (Risk / Non-risk)	P	OR allelic	P	ORAa; ORAA [CI 95%]	P	OR allelic	P	ORAa; ORAA [CI 95%]
1	BICF2S23248027	G/C	0.01	0.64 [0.45–0.92]	0.04	0.76 [0.36–1.59]; 0.42 [0.19–0.93]	0.15	0.76 [0.52–1.11]	0.31	0.75 [0.36–1.55]; 0.54 [0.24–1.22]
	BICF2P468585	C/A	0.02	0.66 [0.46–0.95]	0.07	0.74 [0.35–1.56]; 0.44 [0.20–0.96]	0.07	0.70 [0.48–1.02]	0.14	0.76 [0.37–1.57]; 0.47 [0.21–1.06]
	BICF2P1037296	A/C	0.08	0.72 [0.50–1.03]	0.20	0.76 [0.38–1.53]; 0.53 [0.25–1.12]	0.03	0.65 [0.44–0.95]	0.08	0.66 [0.33–1.32]; 0.41 [0.19–0.90]
	BICF2P357728	G/A	0.08	0.72 [0.50–1.03]	0.19	0.74 [0.37–1.49]; 0.51 [0.24–1.08]	0.03	0.65 [0.44–0.95]	0.07	0.67 [0.34–1.33]; 0.41 [0.19–0.90]
	BICF2S23329752	G/A	0.02	0.62 [0.42–0.92]	0.01	0.33 [0.13–0.85]; 0.26 [0.10–0.66]	0.25	0.79 [0.53–1.18]	0.47	0.99 [0.42–2.31]; 0.71 [0.31–1.63]
	BICF2S23660342	G/A	0.01	0.61 [0.41–0.90]	0.01	0.34 [0.13–0.91]; 0.26 [0.09–0.66]	0.28	0.80 [0.53–1.20]	0.56	0.88 [0.37–2.11]; 0.68 [0.29–1.61]
	BICF2P1129598	C/A	0.03	0.63 [0.42–0.95]	0.05	0.42 [0.14–1.23]; 0.30 [0.11–0.85]	0.16	0.74 [0.49–1.12]	0.39	0.75 [0.29–1.94]; 0.57 [0.22–1.45]
9	BICF2S23027935	A/G	0.26	0.82 [0.58–1.17]	0.23	1.19 [0.62–2.27]; 0.72 [0.35–1.47]	**1.0x10-3**	**0.53 [0.36–0.78]**	**3.8x10-3**	**0.52 [0.27–1.00]; 0.26 [0.12–0.58]**
	BICF2P742007	G/A	0.74	0.94 [0.66–1.35]	0.35	1.50 [0.71–3.16]; 1.05 [0.48–2.28]	**1.0x10-5**	**0.42 [0.29–0.62]**	**8.3x10-5**	**0.35 [0.17–0.74]; 0.17 [0.07–0.39]**
	BICF2G630837240	A/G	0.72	1.07 [0.75–1.52]	0.81	1.22 [0.67–2.22]; 1.13 [0.55–2.32]	**7.9x10-5**	**2.17 [1.47–3.20]**	**4.2x10-4**	**1.97 [0.92–4.20]; 4.78 [2.08–10.98]**

The threshold for significance to correct for multiple testing was determined as 0.05/8 = 6.25x10-3, where the denominator comes from four independent loci multiplied with two phenotypic tests. The significant P-values and corresponding odds ratios are in bold font.

### Top markers on the first locus on chromosome 9 are in linkage disequilibrium and are differentially associated between mildly and moderately-to-severely dysplastic hips

The three most common genotypes for the locus with the strongest association on chromosome 9 in the original GWAS (BICF2S23027935 and BICF2P742007, Table 1) were the homozygous GA (both SNPs represent the non-risk allele) and AG (both SNPs represent the risk allele), and the heterozygous RR (IUPAC coding) (Table 1: BICF2S23027935 and BICF2P742007). The AG genotype differentiates moderate-to-severe from mild hip dysplasia (Table 6). Given the GA and AG genotypes, the odds ratio [95% confidence interval] for mild cases and controls is 0.90 [0.41–2.07], for mild, moderate or severe cases and controls 0.27 [0.16–0.45], and for moderate-to-severe and mild cases 0.16 [0.09–0.29].

**Table 6:** Cross-tabulated frequencies and counts of the most common genotypes of the two top SNPs on chromosome 9 (BICF2S23027935, BICF2P742007) with the hip phenotypes.

Genotype	Hip phenotype			
	Control	Mild	Moderate-to-severe	All cases and controls together
GA	0.40 (100/253)	0.32 (21/66)	0.13 (21/159)	0.30 (142/478)
RR	0.42 (106/253)	0.52 (34/66)	0.48 (76/159)	0.45 (216/478)
AG	0.19 (47/253)	0.17 (11/66)	0.39 (62/159)	0.25 (120/478)
**Total per phenotype group**	1.00 (253/253)	1.00 (66/66)	1.00 (159/159)	1.00 (478/478)

### Identification of additional variants on chromosome 9

To identify additional CHD-associated variants, we resequenced a 7 Mb genomic target (corresponding to bases 30620001–37620000 on chromosome 9) in 24 control and 24 affected dogs representing the most common homozygous SNP genotype combinations (SNPs BICF2S23027935, BICF2P742007, BICF2G630834826, BICF2G630837209, BICF2G630837240, BICF2G630837405 and BICF2P272135; see Tables 1 and 2 and methods). We used a custom pipeline to systematically screen the target area in comparison with the CanFam3.1 annotation. Altogether we found 30197 unique variants in 21140 positions (S2 Table) and classified them based on the associated gene, the predicted functional effect of the variant and the phenotype of the individual. The study design, however, does not permit the direct assessment of the association between the phenotype and the genotype as the case and control animals were selected based on both the phenotype and an opposing homozygous combined genotype of seven top SNPs on chromosome 9 (see Tables 1 and 2 and the methods). We therefore screened for variants that segregated completely or nearly completely with either group of dogs. The difference in counts of each variant between the two groups were determined (S2 Table). 61 variants remained after excluding those displaying an absolute difference of 21 or smaller, those with an intergenic or intronic location, and those leading to a synonymous mutation (S3 Table). An upstream variant was found in the immediate vicinity of *SMG8* encoding for a nonsense mediated mRNA decay factor. However, there is a gap in the dog/human alignment at this position. A DNase I hypersensitivity site and an H3K27Ac signal reside in the human genomic regions homologous to those harboring upstream variants of benzodiazepine receptor (peripheral) associated protein 1 (*BZRAP1*) and ring finger protein 43 (*RNF43*). A strong H3K27Ac signal was also seen in an area on the human chromosome 17 corresponding to a variant upstream of RAD51 paralog C (*RAD51C*). Several potential splicing mutations were seen in *RNF43* and testis expressed gene 14 (*TEX14*). Two variants downstream of *ANKFN1* were in close proximity (within 10 kb) to a SNP with a statistically significant association with the phenotype (S3 Table). See S4 Table, S5 Table, S4 Fig and methods for the calculation of the association between the 217 target area SNPs and the phenotype, N = 426. We also assessed how the variants targeted specific genes so that the target genes segregated with the risk or non-risk SNP genotypes leading to various functional effects (Table 7).

**Table 7:** Variants that segregated between cases and controls categorized by the associated gene and the predicted functional effect.

Gene name	Functional effect							Comments
	downstream	nonsynonymous	exonic;splicing	ncRNA_exonic	splicing	upstream	UTR3	
***AATF***	0	**23**	0	0	0	0	4	rs852180586 (E->K, tolerated), no or low H3K27Ac signal in the corresponding human genomic region in chr.17
***ANKFN1***	**23**	0	0	0	-2	-12	0	No or low H3K27Ac signal in corresponding human genomic region on chr.17, gap in the human/mouse/dog alignment
***MED13***	0	0	0	0	0	0	**22**	No or low H3K27Ac signal in corresponding human genomic region on chr.17
***MPO***	0	**23**	0	0	0	0	0	rs24532262 (S->W, SIFT 0.04 deleterious), not in the mature protein
***PCTP***	**-22**	**-22**	0	0	0	7	0	No or low H3K27Ac signal in corresponding human genomic region on chr.17 and chr.11; missense variant (A216P) outside of any known protein domain on ENSCAFP00000037414.1
***PIGW***	**23**	**23**	0	0	0	0	0	Gap in the human /dog alignment, no functional elements in the corresponding region in mouse chr 11; rs24560601 (I->V, tolerated)
***RAD51C***	-14	0	0	0	0	**23**	-5	Strong H3K27Ac and a nearby DNaseI signal in corresponding human region on chr. 17
***RNF43***	-19	2	**23**	0	**23**	7	0	rs852464446, splicing mutation (33059666) close to SD in intron 1–2 in both transcripts; rs850635127, splicing mutation (33006594) close to SA in intron 3–4 in transcript ENSCAFT00000027763.4 and nonsynonymous variant of transcript ENSCAFT00000043743.1 (P->L, tolerated, low confidence)
***SCPEP1***	-21	0	0	0	-19	3	**-24**	No or low H3K27Ac signal in corresponding human genomic region on chr.17. Gaps in the human/mouse/dog alignment
***SEPT4***	0	-13	0	0	0	**23**	4	No or low H3K27Ac signal in corresponding human genomic region on chr.17
***TEX14***	**23**	**23**	0	0	**23**	2	0	Downstream variants: no or low H3K27Ac signal in corresponding human genomic region on chr.17; rs850849831 (H->R, tolerated), rs851033426 (M->T, tolerated), rs850562371 (N->S, tolerated), rs851547031 (W->L, tolerated), rs850850258 (Q->E, tolerated); splicing mutations in the introns
***U1***	3	0	0	**23**	0	8	0	rs852311430, no overlap with Ensembl Regulatory features or with Ensembl Motif features

The numbers indicate the difference between the numbers of cases and controls carrying variants of a particular functional effect regardless of the position within the indicated gene. Completely or nearly completely segregating variants are in bold. Negative numbers indicate that the variants were more prevalent among the controls. The various variants are commented in the last column. SD = splice donor. SA = splice acceptor. Single letter IUPAC amino acid coding is used. Functional consequences of coding variants have been estimated with SIFT (sift.jcvi.org) and provided by the Ensembl genome browser (www.ensembl.org). The H3K27Ac chromatin marks are from the ENCODE project (www.encodeproject.org) and provided by UCSC Genome Browser (genome.ucsc.edu).

A missense variant rs852180586 in Apoptosis antagonizing transcription factor (*AATF*) is 26.5 kb away of BICF2G630837405 (Tables 2 and 7 and S3 Table) but is predicted to be tolerated. The two variants downstream *ANKFN1* are close to BICF2G630834765 (Table 7, S3 Table) but there is no evidence for a functional effect. The potentially deleterious missense variant rs24532262 in the myeloperoxidase (*MPO*) gene was connected with the cases. The mutation does not target the mature protein, however. A missense variant in *PCTP* corresponds to a location near the carboxy-terminus of the phosphatidylcholine transfer protein that is outside of any known protein domain. All other coding variants were predicted to be tolerated. A potentially regulatory variant was discovered 364 bp upstream of *RAD51C*. This variant was found in 22 cases (16 homozygous, 6 heterozygous animals) and none of the controls. The corresponding site on human chromosome 17 displays a strong H3K27Ac (acetylation of lysine 27 of the histone H3 protein) chromatin mark signal. No other evidence for gene regulatory variants was found. Intronic variants close to splice regions were discovered in the gene for ring finger protein 43 (*RFN43*) and *TEX14*.

### Identification of a regulatory variant upstream of *NOG*

**Fig 4:**
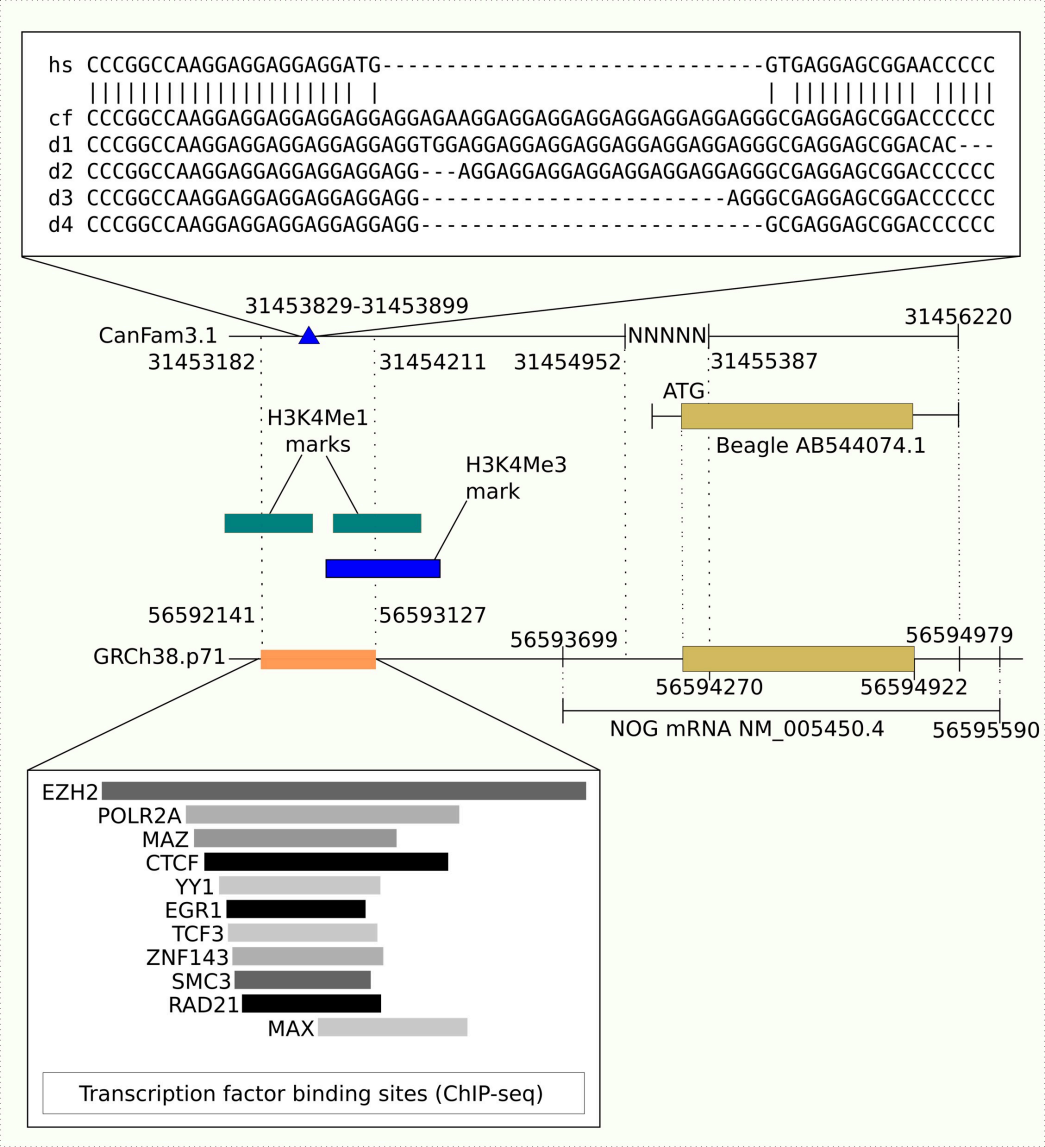
Human *NOG* enhancer region compared to the related region in the dog genome. At the top of the image there is a sequence comparison between human and dog. hs = human genomic (GRCh38.p7) region at chr17:56592775–56592815. cf = canine genomic (CanFam3.1) region at chr9:31453829–31453899. d1-d4 = the corresponding sequences of Dog1-Dog4. Below the sequence comparison is first the canine genomic region including the deletion (blue triangle), gap region marked with Ns, and the complete coding sequence for *NOG* from a Beagle (GenBank: AB544074.1). At the bottom of the image are the corresponding human genomic regions with a TF binding site (orange box) and TFs that can bind to this site as predicted by the ENCODE ChIP-seq experiments. Green boxes are H3K4Me1 histone mark (commonly associated with enhancers) peaks, and the blue box is a H3K4Me3 histone mark (commonly associated with active promoters) peak from the ENCODE data.

Twenty-eight SNPs in the resequenced target region associated with the phenotype (S5 Table). These SNPs concentrated on two loci (S5 Fig, S5 Table) corresponding to those found in the LD-analysis (S1 Table, S3 Fig). As the sequencing depth was variable we combined the reads from cases and controls to separate pools. Visual inspection of two pools of sequences revealed a deletion variant at chr9:31453837–31453860 in the first locus (vertical black line in S5 Fig). This 24-bp deletion variant at resided within an AGG-triplet repeat region in close proximity to the *NOG* gene in eight control dogs. In addition, one dog had a 27 bp deletion. *NOG* and its upstream sequence are conserved across species [33] (S6 Fig). The corresponding region on the human chromosome 17 is placed within a putative gene regulatory element upstream of *NOG* gene with binding sites for several transcription factors [34] (Fig 4). Additionally, there are H3K4Me1 (mono-methylation of lysine 4 of the H3 histone protein) and H3K4Me3 (tri-methylation of lysine 4 of the H3 histone protein) histone mark peaks linked to this region; H3K4Me1 marks associate with enhancers and H3K4Me3 with active promoters [34] (Fig 4). The corresponding region on mouse chromosome 11 overlaps with binding sites for Suz12 (OREG1916695) and Mtf2 (OREG1828914) transcription factor binding sites.

### *NOG* variant associates with normal or mildly affected hips but not with moderate-to-severe hip dysplasia

The presence of the deletion variant upstream of *NOG* (Fig 4) was directly assessed by PCR in the whole population of dogs in this study. The fragment sizes were analyzed by gel electrophoresis for all the samples. PCR failed to give a product in nine samples and the product was ambiguous in one sample. The deletion genotype counts and frequencies for each phenotype category for the remaining 516 dogs are shown in Table 8.

**Table 8:** Number of upstream *NOG* deletion genotypes in cases and controls.

Genotype	Phenotype group				
	Mild cases	Moderate-to-severe cases	All cases together	Controls	All cases and controls together
**No deletion**	0.29 (23/79)	0.60 (98/164)	0.50 (121/243)	0.37 (101/273)	0.43 (222/516)
**Deletion in one allele**	0.57 (45/79)	0.35 (57/164)	0.42 (102/243)	0.47 (127/273)	0.44 (229/516)
**Deletion in both alleles**	0.14 (11/79)	0.05 (9/164)	0.08 (20/243)	0.16 (45/273)	0.13 (65/516)
**Total per phenotype group**	1.00 (79/79)	1.00 (164/164)	1.00 (243/243)	1.00 (273/273)	1.00 (516/516)

### *NOG* variant correlates with and improves the predictive power of SNP genotypes

The deletion genotype correlated with the genotypes of the SNPs BICF2P742007 and BICF2S23027935 in all three phenotype categories. Spearman’s rank correlation coefficient rho [with 95% confidence intervals] was 0.64 [0.56–0.71] for controls, 0.69 [0.56–0.79] for mild cases and 0.67 [0.58–0.75] for moderate-to-severe cases (S6 Table). The significance of the protective effects of the *NOG* deletion and GA SNP genotype in various subsets of dogs was next investigated using logistic regression. The odds ratios for the corresponding generalized linear model (GLM) coefficient estimates are presented in Table 9. In contrast to the effect of the GA SNP genotype, the protective effect of the *NOG* deletion was most significant between the mild and moderate-to-severe cases (Table 9).

**Table 9:** The odds ratios based on logistic regression modeling of the CHD phenotype using the *NOG* deletion allele and the GA top SNP combination genotype as independent variables.

Subset	OR [95% confidence interval]		
	Deletion in both alleles	Deletion in one allele	GA SNP genotype
Controls (N = 272); all cases (N = 243)	0.74 [0.36–1.51]	0.81 [0.55–1.19]	0.41 [0.25–0.68]
Controls (N = 272); moderate-to-severe cases (N = 164)	0.43 [0.17–1.03]	0.56 [0.36–0.87]	0.36 [0.19–0.64]
Mild cases (N = 79); moderate-to-severe cases (N = 164)	0.24 [0.07–0.78]	0.31 [0.17–0.58]	0.74 [0.33–1.69]

To assess the effect of the deletion, we compared the full (with the *NOG* deletion) and the reduced (without the *NOG* deletion) GLM models using chi-squared test. There was a statistically significant difference between the full and reduced models on controls and moderate-to-severe cases (P < 0.05) and on mild and moderate-to-severe cases (P < 0.001, Table 10). Finally, the receiver operating characteristic curve was used to assess the discrimination potential between the full and reduced models. We argue, based on the results from this comparison, that the full rather than reduced model better discriminates the controls and moderate-to-severe cases (P < 0.01), as well as the mild and moderate-to-severe cases (P < 0.001, Table 10).

**Table 10:** Comparison of full (with *NOG* deletion) and reduced (without *NOG* deletion) generalized linear models on three subsets of dogs.

Subset	Model	Residual Df	Residual Deviance	Df	Deviance (P)	AUC	Z (P)
Controls (N = 272); all cases (N = 243)	Full	511	686.76	2	1.37 (0.50)	0.61	1.31 (9.5x10-2)
	Reduced	513	688.13			0.60	
Controls (N = 272); moderate-to-severe cases (N = 164)	Full	432	538.63	2	7.88 (1.9x10-2)	0.66	2.71 (3.3x10-3)
	Reduced	434	546.51			0.62	
Mild cases (N = 79); moderate-to-severe cases (N = 164)	Full	239	284.68	2	15.09 (5.3x10-4)	0.66	3.59 (1.6x10-4)
	Reduced	241	299.77			0.57	

Df = degrees of freedom, P = probability value, Z = Z test statistic, AUC = area under the receiver operating characteristic curve

### The deletions upstream of *NOG* downregulate reporter gene expression *in vitro*

We investigated the effects of the deletions on the expression of a luciferase reporter gene *in vitro*. We designed three constructs (S7 Table), where the longest construct A with 14 AGG-triplet repeats corresponds to resequencing data from Dog2 (S7 Fig and Fig 4). Construct B had a deletion of eight AGG-triplets, and construct C had a deletion of seven AGG-triplets when compared to construct A (Fig 4). The sequences corresponding to the constructs A and C were common in the cohort, whereas we recovered the sequence corresponding to variant B in only one individual. The constructs were cloned to a plasmid containing a luciferase reporter under the control of a minimal promoter. We used two experimental setups with HEK293 human embryonic kidney and U-2 OS human osteosarcoma cell lines: HEK293 cells with 50 ng and U-2 OS cells with 10 ng DNA.

**Fig 5:**
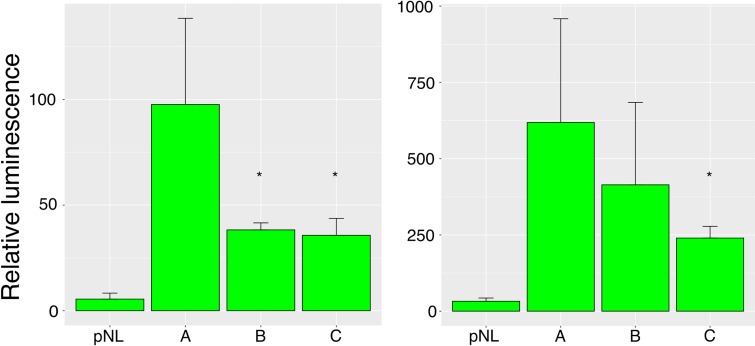
The differential effects of three *NOG* regulatory alleles in a dual luciferase reporter assay. Relative luminisence (NanoLuc/firefly luminisence expressed as median value ± standard deviation, three biological replicates with four technical replicates each per every construct). Left: HEK293 cells transfected with 50 ng plasmid DNA + 50 ng carrier DNA. Right: U-2 OS cells transfected with 10 ng plasmid DNA + 10 ng carrier DNA. pNL: empty control vector. A: construct A, B: construct B, C: construct C. *: P<0.05 relative to construct A. The difference between relative luminescence of the control plasmid and each of the constructs was always statistically significant.

The results are expressed as mean ± SD of four technical replicates from three independent experiments for each cell line and treatment. The firefly luminescence control was used to normalize the NanoLuc luminescence values. In the first experimental setup with HEK293 cells, construct A had significantly higher luminescence compared to both B and C constructs (Fig 5). Again, in the second setup, with U-2 OS cells, the A construct demonstrated significantly higher luminescence than construct C. All comparisons between the control plasmid and construct luminescence levels were significant.

### Genome sequence for the canine *NOG* locus

The current canine reference genome (CanFam3.1) shows a gap within *NOG* (Fig 4). We closed the gap by PCR and sequencing (S8 Fig). The sequence overlapped the 5’ *NOG* sequence from Beagle [35] and corresponded with the variant with three copies of hexanucleotide insertion (GenBank accession AB544074.1). The closure of the gap in the reference genome permits the accurate positioning of the upstream deletion locus in relation with the coding sequence. The sequence corresponding to the 434 bp long gap in the reference is very similar to the corresponding human sequence (S9 Fig). Alignment introduced six gaps (34, 25, 3, 2, 1 and 1 bp). The nucleotide-level identity was a remarkable 76% (330/434) suggesting conserved function. The *NOG* promoter (ENSR00000096009) overlaps with the corresponding region in human and spans from 17:56592202 to 17:56594999 with the core promoter at 56592600–56594601. Scanning the human core collection at the JASPAR2018 database [36] with the alignment of dog and human sequences at S9 Fig, uncovered 135 matrix IDs and altogether 1368 putative binding sites for them (S8 Table). Bonferroni-adjusted P-values were calculated for all sites. Matrix IDs with adjusted P-value less than 0.05 for any site are shown in S9 Table. The corresponding transcription factors are histone 4 transcription factor (HINFP), two E2F-related factors and three AP-2 family members.

## Discussion

Canine and human hip dysplasia represent one of the most complex and prevalent problems in veterinary and medical sciences. Our GWAS uncovered four novel protective and risk loci on chromosomes 1 and 9. The loci on chromosome 9 differentiated the mild from the moderate-to-severe phenotypes. Alleles upstream of *NOG* displayed differential enhancer activity *in vitro*. Three additional candidate genes on chromosomes 1 and 9 were revealed: *NOX3*, *ARID1B* and *RNF43*.

We identified putative regulatory variants of *NOG* that encodes for a well-known BMP inhibitor, noggin. Noggin is essential for the growth and patterning of the neural tube after neural induction [37,38], but it is also required for embryonic chondrogenesis, osteogenesis and joint formation [38–40]. Joint formation in *Nog* knockout mice is defective and most joints are missing from the limbs [40]. In humans, *NOG* missense mutations segregate with proximal symphalangism and multiple synostosis syndrome, both of which are skeletal dysplasias resulting from decreased noggin activity [39,41]. *Nog* is also widely expressed in adult mouse joint cartilage and down-regulated in surgically induced arthritis [42]. *Nog* haploinsufficiency protected mice from arthritis induced by methylated bovine serum albumin [43]. Overexpression of murine noggin has been associated with impaired function of osteoblasts, resulting in osteopenia, fractures and decreased bone formation rate [38,44,45].

The affinity of noggin to different BMPs varies. Further, there are other BMP antagonists that can partially compensate for the lack of noggin (e.g. chordin, follistatin, gremlin and sclerostin) [37,40,46–48]. However, siRNA-mediated Nog knock-down led to increased BMP-mediated osteoblastic differentiation and extracellular matrix mineralization without compensatory induction of gremlin or chordin expression [49]. Our *in vitro* expression data suggests that the variant upstream of *NOG* has potential gene-regulatory consequences. It is possible that the regulation of noggin expression levels is suboptimal in hip joints of German Shepherds prone to develop moderate-to-severe hip dysplasia. Another study revealed a single-nucleotide variant affecting the expression of *NOG* 105 bp downstream of the transcription start site, when the researchers investigated targeted sequencing data of a GWAS locus for human cleft lip, with or without cleft palate [50].

We were not able to close the 434 bp gap upstream of *NOG* with the targeted resequencing data. The overall coverage was variable, and parts of the target region were not covered at all. This is a general caveat of using probe-enriched genomic DNA templates for sequencing. We finally used PCR and sequencing to close the gap, which enabled the accurate positioning of the upstream deletion locus in relation with the coding sequence. The close proximity with *NOG* and the high degree of conservation with the corresponding sequence in human *NOG* promoter suggest that the uncovered new genomic sequence might be involved in the regulation of *NOG* expression. Together with the discovery of functionally active variant alleles upstream of *NOG* (Figs 4 and 5), our results suggest more research should be targeted to the characterization of canine *NOG* and its regulation.

The protective locus on chromosome 1 spans over a 1.1 Mb region and harbors two genes of interest: *NOX3* and *ARID1B*. NOX3 belongs to the family of NADPH oxidases, which catalyse the formation of superoxides and other reactive oxygen species. NADPH oxidase enables the production of hydrogen peroxide (H_2_O_2_), which is ultimately used in a reaction cascade that participate in the initiation of articular cartilage degradation [51,52]. *NOX3* is a non-phagocytic member of the NADPH oxidase family and it is mainly expressed in the inner ear and fetal tissues [53]. Thus, the role of NOX3 molecule in hip dysplasia remains uncertain, although as shown in S10 Table, an indirect link between NOX3 and TRIO, a protein encoded by another candidate gene for German Shepherd hip dysplasia has been reported in a study by Fels et al. (2014) [54].

The AT-rich interactive domain-containing protein 1B encoded by the second candidate gene (*ARID1B*) on chromosome 1, functions as a transcriptional activator and repressor via chromatin remodeling [55]. Mutations in *ARID1B* cause Coffin-Siris syndrome (CSS), which is a rare hereditary disorder affecting multiple body systems, for instance the nervous, cardiovascular, and skeletal systems [56,57]. As a consequence to this syndrome, *ARID1B* is associated with joint laxity (66% of the patients) [56,57]. However, the dogs with hip dysplasia do not exhibit similar multisystemic symptoms as the CSS patients with causative *ARID1B* mutations. *MAMDC2* is another potential candidate gene on chromosome 1. It encodes a proteoglycan and has been associated with increased intraocular pressure [58].

Other putative candidate genes on chromosome 9 uncovered in the variant analysis (Table 7) include *MPO*, *RNF43*, *RAD51C*. Reactive oxygen species and MPO have been inferred to participate in the regulation of chronic inflammation [59–61]. Therefore, it was intriguing to discover a potentially deleterious missense variant of *MPO* (Table 7). The mutated amino acid, however, is not included in the predicted mature protein. Intronic variants close to splice regions in *RNF43* are also potentially significant. RNF43 ubiquitin ligase [62] negatively regulates WNT signaling [63]. WNT signaling is implicated in osteoarthritis as reviewed in [64,65], and a recent study also suggest it might be affected in CHD [66]. RAD51C is a well-known recombination factor [67].

Deciphering polygenic, multifactorial disorders requires large sample sizes. Although dogs have an unique genomic architecture [28,68–70] that facilitates association studies in smaller cohorts than in humans [28], the lack of power is still a regular concern. Our GWAS was unexceptional in this respect. Even after we increased the sample size from 292 to 409 or 524 dogs, and consequently revealed two additional loci on chromosome 1, none of the associations reached genome-wide significance. We observed strong LD in our data (S1 Table), which was expected due to the genomic architecture of dogs. Therefore, Bonferroni correction threshold could be overly conservative for our data as explained in Methods, part “Genome-wide association analysis”. Also, the lack of power may be a consequence of the increasing variation among cases, when we included the mild hip dysplasia phenotypes in the second meta-analysis. We observed significant differences in the allele and genotype frequencies between mild cases (C/C) and the moderate-to-severe cases (C/D, D/C or worse) throughout the loci on chromosome 9 (Table 5), whereas mild cases did not differ from controls in these comparisons. Additionally, the fragment genotype frequencies related to *NOG* were similar for controls and mild cases but again differed significantly when these two groups were separately compared with the moderate-to-severe cases. These findings corroborate that the dogs with mild hip dysplasia are indeed at lower genetic risk for the disorder. It would be important to find out, if other genetic factors differentiating the dogs in these phenotype groups exist.

In conclusion, using several genetic approaches we have discovered novel variants of a putative *NOG* enhancer that downregulate reporter gene expression *in vitro*. The variants are associated with healthy and mildly dysplastic hip joints in German Shepherds. Besides a larger replication study and investigation of the other candidate genes on chromosomes 1 and 9, future research should focus on what kind of biological effects the variants have on the expression of noggin in the canine hips and on the development of hip dysplasia.

## Methods

### Animals

The Finnish Kennel Club (FKC) granted permission to use its data and CHD screening radiographs for our research. All radiographs have been scored by two specialized veterinarians, thus reducing inter-observer bias [7]. All hip score results are freely available from the FKC breeding database [71].

Our study cohort consisted altogether 531 German Shepherds (247 cases + 284 controls), born between 1993 and 2013. Cases were dogs with an FCI score C or worse for both hips and controls were dogs with a score A for both hips. We discarded dogs with an FCI score B because their inclusion may lead in a confounded control phenotype. Five control dogs had to be excluded from the analyses due to ambiguous phenotypes. This left us with a total of 526 dogs (247 cases and 279 controls) before quality control. However, one more control had to be removed during quality control due to an outlier genotype, after which we had 525 dogs left for the GWAS.

At least one EDTA blood sample was collected from all the dogs between years 2006 and 2015. The dogs were chosen for our study according to their hip scores and pedigrees, creating a balanced study population of working, mixed and show line dogs (S10 Fig).

### Ethics

Guidelines for research ethics and good scientific practices were followed. We hold an ethical license for collecting EDTA blood samples (ESAVI/7482/04.10.07/2015), from ELLA–Animal Experiment Board in Finland under The Regional State Administrative Agency for Southern Finland. The owners signed a form of consent and they were well informed of the project.

### DNA preparation and genotyping

The original EDTA-blood samples are stored at the Dog DNA bank at the University of Helsinki. DNA extraction from the EDTA-blood samples was carried out using Chemagic Magnetic Separation Module I (MSMI) with a standard protocol by Chemagen (Chemagen Biopolymer-Technologie AG, Baeswieler, Germany), after which the samples were sent to Geneseek (Lincoln, NE, US) to be genotyped using the high density 173K canine SNP array from Illumina (San Diego, CA, USA). Genotyping was executed in several batches as collection of the original EDTA-samples took place over several years. Batch effect was accounted for as a covariate in our meta-analyses.

### Population structure

Our German Shepherd population was divided into five (the original GWAS, S11 Fig) or four (meta-analyses, S12 Fig) subpopulation clusters according to their genomic relationships. This was achieved by first calculating the appropriate number of clusters from a genomic relationship matrix with a package “mclust” [72] in R [73], which uses covariance parametrization and selects appropriate clusters via Bayesian information criterion. A covariate vector was created according to the clustering data, so that each individual belongs to one of the clusters. This covariate was used in our model to account for any differences in disease association between the clusters.

### Quality control (QC)

#### Original GWAS

Initial merging of the genotype sets and a genotype missingness test was performed with PLINK [74]. 6058 SNPs failed the missingness test with a threshold of 0.05. In total, 166309 SNPs and 293 samples were transferred from PLINK to R. We performed the final QC with the following thresholds: minor allele frequency = 0.05, per sample call rate = 0.90 and per SNP call rate = 0.95, p-value cut-off level < 0.00001 to test for deviations from Hardy-Weinberg equilibrium (HWE). The HWE-check was executed on controls as cases may show deviation from HWE in association with the disease [75]. The QC resulted in the final data of 92315 autosomal SNPs and 293 samples. However, one sample was manually removed after the check.marker-function due to an outlier genotype, which left 292 samples for our association analysis. The position map for our SNPs was CanFam3.1. After the GWAS, we checked the genotype call quality of the best SNPs to verify that the associations were not due to genotype-calling errors.

### Meta-analyses

The same genotype data was used for both meta-analyses, but the number of included dogs in each analysis was determined by the stringency of the phenotypes. The quality control for this data was carried out in two steps. First, before merging the original data (292 dogs) with the new genotypes (233 dogs), initial quality controls were executed separately on them with PLINK. The following thresholds were used for each data set: per sample call rate 0.90, per SNP call rate 0.95, minor allele frequency 0.05, and p-value cut-off level < 0.00001 for the HWE check. Also, strand had to be flipped for 59980 SNPs in the original data set due to strand inconsistencies with the new genotype data. This was done with the --flip command in PLINK. Second, after merging of the data sets, the data was imported to R, where the QC was repeated with GenABEL for the whole data with the same QC thresholds. This left 88499 SNPs for the meta-analyses. After the meta-analyses, we checked the genotype call quality of the best SNPs to verify that the associations were not due to genotype-calling or other such errors. One SNP on chromosome 4 (BICF2P491963) was observed to show false association in the first meta-analysis due to a batch-specific calling error. The error was not resolved by the use of batch covariates, and the SNP was therefore removed. Also, the genotyping batch of one dog was missing. This dog was therefore removed leaving 524 dogs for the meta-analyses.

### Genome-wide association analysis

We performed a case-control GWAS to identify SNPs associated with canine hip dysplasia. The original association study included 160 controls and 132 cases. The GWAS was implemented in R with the package GenABEL [31]. The covariates were sex and the genomic cluster of the animal. In the meta-analyses we also used the genotyping batch as a covariate. We used FASTA [76] and QTSCORE [32], in GenABEL to calculate the association test statistics. When used with a binary trait FASTA corresponds to the Cochran-Armitage trend test [77].

FASTA is an efficient tool for association analysis in family-based data sets. However, FASTA has the disadvantage of not being able to compute a genome-wide significance with permutation analysis, because the data structure of the test statistics is not exchangeable. This is due to incorporating the relationship matrix ϕ in the computation of the test statistics [76]. QTSCORE does not suffer from this, as the test statistics derive from the environmental residuals that are not correlated with each other. Thus, the data structure is exchangeable and permutation analysis can be used to calculate empirical experiment-wise genome-wide significance levels for the analyzed SNPs [32].

Bonferroni correction threshold for genome-wide significance was determined as (P-value/Number of SNPs) = 0.05/92315 = 5.42x10-7 for the original GWAS, and 0.05/88499 = 5.65x10-7 for the meta-analyses. However, Bonferroni correction is problematic in genetic association studies, because it expects independence between the comparisons, which does not hold for SNPs due to LD [78]. Consequently, when type I error is controlled with overly conservative Bonferroni adjustment, type II error rate might be inflated if the sample size is small, and some QTL with real effects may be ruled insignificant [78]. Therefore, we estimated the effective number of independent tests using simpleM [79] for use in permutation analysis for genome-wide significance as 24159 for the original GWAS and 26323 for the meta-analyses. We also used these values to calculate thresholds for significance that rely on more accurate estimates of independent tests: 0.05/24159 = 2.07x10-6 for the original GWAS and 0.05/26323 = 1.90x10-6 for the meta-analyses.

### Assessment of linkage disequilibrium and number of independently associated loci

We used the function “r2fast” [80] from the GenABEL-package in R to estimate the r^2^ values between the top SNPs from the genome-wide association analyses. For one SNP in the first meta-analysis (BICF2P272135), we re-calculated the r^2^ values with the RSQ-function in excel, because of a batch specific allele flip that affected the LD-estimation in R. To estimate the number of independently associated loci within the target regions on chromosomes 1 and 9, we used a SNP clumping procedure. This was executed with the “clump.markers” function from the R-package cgmisc [81]. The threshold for forming the clumps were as follows. The physical distance cut-off for clumping was set to 7.5 Mb to cover all of the associated loci on both targeted chromosomes, so as not to create any clumps due to distance, but only due to association with the trait (P-value threshold = 5.0x10-5–5.0x10-6), and due to high enough correlation between the SNPs (r^2^ threshold = 0.70).

### Targeted sequencing

A targeted sequencing of a 7 Mb region on canine chromosome 9 (bases 30620001 to 37620000 from NC_006591.3) was executed by the DNA Sequencing and Genomics lab at the University of Helsinki. The study included 24 cases and 24 controls that were chosen by the combinations of their genotypes for the following markers: BICF2S23027935, BICF2P742007, BICF2G630834826, BICF2G630837209, BICF2G630837240, BICF2G630837405 and BICF2P272135 (See also Tables 1 and 2). SNP genotype combinations for 24 controls and 23 cases were GAGAGCG and AGAGATC, respectively. In addition, one case had the combination AGARRYS. An indexed Illumina library was created for all 48 samples. Briefly, DNA was sheared using a Bioruptor NGS sonicator (Diagenode, Denville, NJ, US) and the obtained fragments were end-repaired, A-tailed and truncated Illumina Y-adapters ligated. In a PCR step (20 cycles) full-length P5 and indexed P7 adapters were introduced using KAPA Hifi DNA Polymerase (KAPA Biosystems, Wilmington, MA, US). Pools containing four samples each were made for sequence capture with custom SeqCapEZ probes (Nimblegen/Roche, Madison, WI, US) targeting the 7 Mb area from the genome. The sequence capture was performed according to the manufacturer’s protocols (Nimblegen/Roche, Madison, WI, US). The captured fragments were amplified (20 cycles) using Illumina adapters P5 and P7 as described above. The PCR products were purified, and size selected using AMPure XP beads (Beckman Coulter Inc., Brea, CA, US). The obtained final libraries were paired-end (300 bp + 300 bp) sequenced on a MiSeq Sequencer (Illumina, San Diego, CA, US). The adapter sequences were removed and the raw reads were filtered using PRINSEQ [82]. After quality control, the remaining 47272947 (94.6%) reads were mapped to the reference sequence CanFam3.1 using Burrows-Wheeler Alignment tool [83]. The aligned reads were visualized in Tablet and Integrative Genomics Viewer [84,85].

### Variant analysis

We implemented a targeted re-sequencing analysis pipeline to screen for coding variants in comparison with CanFam3.1 reference genome. FASTX was used to perform base quality check of the raw reads and Burrows-Wheeler Aligner (BWA) version 0.5.9 [83] was used to map the reads to the reference genome. Picard tools (http://broadinstitute.github.io/picard/) was used to sort and mark possible PCR duplicates. Re-alignment around indels and base quality score recalibration was done using GATK. The variant calling was carried out using the Genome Analysis Tool Kit (GATK) version 3.5 [86] and SAMtools version 1.2 [87,88]. The detected variants were annotated to Ensembl and NCBI gene annotation databases using ANNOVAR [89].

Using 258 controls (hip scores A/A, including the sequenced 24 controls) and 168 moderate-to-severe-cases (hip scores C/D, D/C or worse, including the 24 sequenced cases), we determined the statistical association between the phenotype and SNP variants in the target area (S4 Table). The Cochran-Mantel-Haenszel test variable M^2^ for the independence of variants and the phenotype could be determined for 217 SNPs (S5 Table). The null distribution of maximum M^2^ from 10000 permutations had a mean value of 8.25 and with 95% confidence interval ranging between 4.06 and 15.01. Using the null distribution as a reference, 28 of the 217 SNPs were statistically associated with the phenotype (Bonferroni-corrected, adjusted p-value < 0.05, N = 217) (S5 Table, S4 Fig).

### Fragment analysis

We performed a PCR with a region of 400 bp encasing the deletion revealed in the targeted sequencing. We designed the primers for this with the NCBI Primer-BLAST tool [90]. The primer sequences are in the supporting information (S11 Table). Basic and 5’-FAM-labeled primers were from Oligomer (Helsinki, Finland). The annealing temperatures were calculated with Thermo Fisher Scientific Tm calculator for Phusion DNA polymerase [91]. The PCR was run with a T100 Thermal Cycler (Bio-Rad, California, US) with a standard 3-step protocol for Phusion reaction. Standard 1.2% and 2% agarose gels were used (A9539; Sigma Aldrich, St. Louis, MO, US), with 1 x TBE buffer and ethidiumbromide staining. Sample and ladder volume were 5 μl in all lanes. We used GeneRuler 100 bp (SM0242) and 100 bp Plus (SM0321), from Thermo Fischer Scientific (Waltham, MA, US) as the DNA ladders. The gel-imaging was performed with AlphaImager (Alpha Innotech, Kasendorf, Germany). The PCR amplicon was validated with sequencing. PCR products from 18 dogs were ambiguous on gels and were sent for fragment analysis. Nine samples did not yield a product with either method and one sample remained ambiguous leaving us with 516 fragment genotypes. The DNA Sequencing and Genomics lab at the University of Helsinki carried out both the sequencing and the fragment analysis. They used capillary electrophoresis to analyze the fragments, with a GeneScan 500 ROX dye (4310361; Thermo Fisher Scientific, Waltham, MA, US) size standard. Subsequently, we analyzed the data with Peak Scanner v1.0 (Applied Biosystems, Foster City, CA, US).

### Logistic regression models

Logistic regression models with or without the *NOG* regulatory variants were computed in R [73]. The odds ratios corresponding with the GLM coefficients were calculated using R package ‘oddsratio’ [92]. AUC calculations and comparisons were done using R package ‘pROC’ [93].

### Assembly of the resequencing data

A reference sequence was assembled using CSC computational hub based on the targeted sequencing reads from a case that did not exhibit the deletion upstream of *NOG*. The adapters were removed and quality of the fastq files was assessed using FastQC [94]. The *de novo* assembly was done using the Spades assembler [95]. Assembly was done for the following k-mer values (21, 33, 55, 77, 99, 127); the Spades assembler then generates a combined assembly (i.e. scaffolds) based on the kmers used. The assembly QC for the scaffolds was done using ‘Quast’ [96].

### Closure and characterization of the gap upstream of *NOG* in the CanFam3.1. reference genome sequence

Genomic DNA from Dog6 was amplified using primers Can*NOG*-F1 and Can*NOG*-R1 from Ishii et al. [35]. The PCR products were sequenced, low quality sequences were discarded and a consensus sequence was derived. The alignments between Dog6, CanFam3.1 chr9 and GRCh38 chr17 were done using MAFFT [97]. The human core JASPAR2018 database [36] was queried with the alignment in S9 Fig using TFBSTools [98]. Bonferroni correction was used to adjust the P-values for each putative binding site for all the matrix ID’s. The matrix ID specific prediction was considered significant if the bonferroni-corrected P-value for any of its binding sites was less than 0.05.

### Dual luciferase reporter assay

According to the findings from the targeted sequencing we designed three different sequence variant constructs: A, B and C, where A is our German Shepherd reference sequence, and B and C are variants with deletion of eight or seven AGG-triplets. The construct sequences are shown in the supporting information (S7 Table). The longest construct (construct A) was designed based on the Dog2 scaffolds generated from the resequencing data (S7 Fig). The *NOG* enhancer sequence variants were cloned into the pNL3.1[*Nluc*/minP] NanoLuc luciferase vector (Promega, Madison, WI, US). pGL4.54[luc2/TK] firefly luciferase was used as a constitutively expressed control plasmid. 24 h prior to transfection, 2 x 10^4^ HEK293 or 8 x 10^3^ U-2 OS cells were plated to 96 well plates in DMEM medium supplemented with 10% FBS and without antibiotics. The HEK293 cells were transfected with 50 ng of each plasmid DNA and 50 ug carrier DNA / well and the U-2 OS cells with 10 ng of each plasmid and 80 ug carrier DNA/well using Fugene HD transfection reagent (Promega, Madison, WI, US). Luciferase activities were measured after 24 h using the Nano-Glo Promega Dual-Luciferase reporter assay system according to the manufacturer’s instructions. The NanoLuc luminescence values were normalized by division with the control firefly luminescence. The data for every setup (three transfection experiments each with four technical replicates) was analyzed in R using the Kruskal-Wallis rank sum test followed by Dunn’s test for multiple pairwise comparisons with Bonferroni adjustment for P-values. P-value < 0.05 was considered significant.

## Supporting information

S1 Fig Q-Q plots with different analysis methods: FASTA and meta-analysis with FASTA, QTSCORE and meta-analysis with QTSCORE. (PDF)

S2 Fig Assessment of the number of independent loci within the targeted region on canine chromosome 9 with a SNP clumping procedure for the original GWAS. Yellow = first locus near *NOG*. Green = second locus near *LHX1*.
(TIFF)

S3 Fig Assessment of the number of independent loci within the targeted regions on canine chromosomes 1 and 9 with a SNP clumping procedure for the GWAS meta-analyses. Left panel: yellow = first locus near *NOX3* and *ARID1B*, green = second locus near *MAMDC2* and *PTAR1*. Right panel: Yellow = first locus near *NOG*. Green = second locus near *LHX1*.
(TIFF)

S4 Fig The null distribution of the maximum value of the M^2^ test variable. The M^2^ test variable for each SNP was calculated from a permutated cohort in S4 Table. The distribution of the maximum value of M^2^ from 10000 permutations is indicated. Vertical lines indicate the mean value and its upper and lower bound with 95% confidence interval. The dotted line indicates the value of M^2^ = 24.10 corresponding to a Bonferroni-adjusted p-value of 0.05. Bandwidth = 0.5.
(PDF)

S5 Fig Densitogram of the statistically significant SNPs along the resequenced target area. Bandwith = 700 kb. The positions of bases on chromosome 9 are indicated on the x-axis. The green vertical lines indicate the positions of the 28 SNPs that associate with CHD. (See the bolded lines in S5 Table) The black vertical line indicates the position of the deletion upstream NOG at 31453837–31453860.
(PDF)

S6 Fig Manually adjusted multiple alignment of the triplet-repeat region upstream of *NOG* from 15 species representing primates, lagomorphs, canidae and felidae. The sequences are from Ensemble, version 94. The stars indicate the insert in Fig 4 spanning over GRCh38 17: 56592775–56592815. This region corresponds to the 5’ *NOG* core promoter (ENSR00000096009 in Ensembl v.94 [33]) in the human chromosome 17: 56,592,600–56,594,601 (GRCh38.p12). The prealigned sequences from 26 eutherian mammals with the human NOG core promoter can be found at: http://oct2018.archive.ensembl.org/Homo_sapiens/Share/cea4ebce4ec4fa397e367c647cf86f8f?redirect=no;mobileredirect=no.
(PDF)

S7 Fig Multiple alignment of construct A, Spades scaffolds for Dog2, Dog1 and Dog5, and the CanFam3.1 reference sequence. The position for the last nucleotide in the alignment is indicated for construct A and the CanFam3.1 chromosome 9 reference (NC_006591.3). Differences among any of the sequences are indicated by asterisks (*). Differences between construct A and CanFam3.1 are painted yellow.
(PDF)

S8 Fig Multiple sequence alignment of the sequence of the gap-closing PCR product (Dog6), chromosome 9 reference (NC_006591.3) and 5’-sequence from the Beagle *NOG* locus (AB544074.1). (PDF)

S9 Fig Alignment between Dog6 and human sequences bridging the 434 bp gap in the dog reference. For clarity, the artificial numbering of the sequence from Dog6 corresponds the location of 434 bp gap on the chromosome 9 sequence (NC_006591.3).
(PDF)

S10 Fig Multidimensional scaling plot for cases and controls within the whole study population. Cases (representing either mild, moderate or severe hip dysplasia) are marked with yellow and controls are marked with black. This figure includes all of the 525 dogs used in the association analyses. The right-hand cluster in this figure consist of working line dogs and the left-hand cluster of show line dogs; mixed line animals are between these two main groups (dogs whose ancestors have both show and working line animals). The separate cluster above the working line cluster consists of a group of closely related dogs (full- and half-siblings and their common female ancestor), and a dog sharing multiple different common ancestors with this family-group.
(TIFF)

S11 Fig Classification of the subpopulations (N = 292) in the original GWAS with the R-package mclust. Best model by mclust: spherical, varying volume with 5 components.
(TIFF)

S12 Fig Classification of the subpopulations (N = 525) in the GWAS meta-analyses with the R-package mclust. Best model by mclust: ellipsoidal, equal shape with 4 components.
(TIFF)

S1 Table Linkage disequilibrium between the top SNPs from the GWAS, described as r^2^ values. (XLSX)

S2 Table Position of 30197 sequence variants, their predicted functional effect and distribution between cases and controls. (XLSX)

S3 Table A subset of 61 variants displaying complete or near segregation with phenotypes. Intergenic, intronic and synonymous coding variants were excluded. Column L indicates the distance to the closest SNP that associates with the phenotype.
(XLSX)

S4 Table The structure of the cohort for analyzing the association between 217 target area SNPs and the phenotype. Distribution of the dogs (N = 426) among cases and controls and the various batches of the SNP genotype analysis is indicated. The Cochran-Mantel-Haenszel test does not accept cells with zero elements, so some dogs were left out.
(XLSX)

S5 Table Association of 217 target area SNPs with the phenotype. Position, value of M^2^ test variable, raw and Bonferroni-adjusted p-values for each SNP. SNPs displaying a statistically significant association (adjusted p-value < 0.05) with the phenotype are in bold font.
(XLSX)

S6 Table Fragment and SNP genotypes in different phenotype categories of 515 dogs. (XLSX)

S7 Table Construct sequences for the Dual Luciferase reporter assay. (PDF)

S8 Table Putative transcription factor binding sites in the sequence corresponding to the 434 bp gap in the dog reference. The human core JASPAR2018 database was queried with the alignment of dog and human sequences in S9 Fig.
(TXT)

S9 Table Tops hits for the transcription factors that are predicted to bind to the sequence corresponding to the 434 bp gap in the dog reference. The human core JASPAR2018 database was queried with the alignment of dog and human sequences in S9 Fig. The matrix ID’s for which the bonferroni-corrected P-value for any binding site is less than 0.05, are presented together with the corresponding transcription factors.
(PDF)

S10 Table Possible indirect interactions between NOX3 and TRIO as suggested by querying the STRING database at string-db.org. (XLSX)

S11 Table Primer sequences for the fragment analysis. (PDF)
