# Supplementary material for: Novel protective and risk loci in hip dysplasia in German Shepherds
Source: PLoS Genet. 2019 Jul 19;15(7):e1008197. doi: 10.1371/journal.pgen.1008197 (PMC6668854; doi:10.1371/journal.pgen.1008197)
Supplement: S1 Fig — (PDF) [file pgen.1008197.s001.pdf]

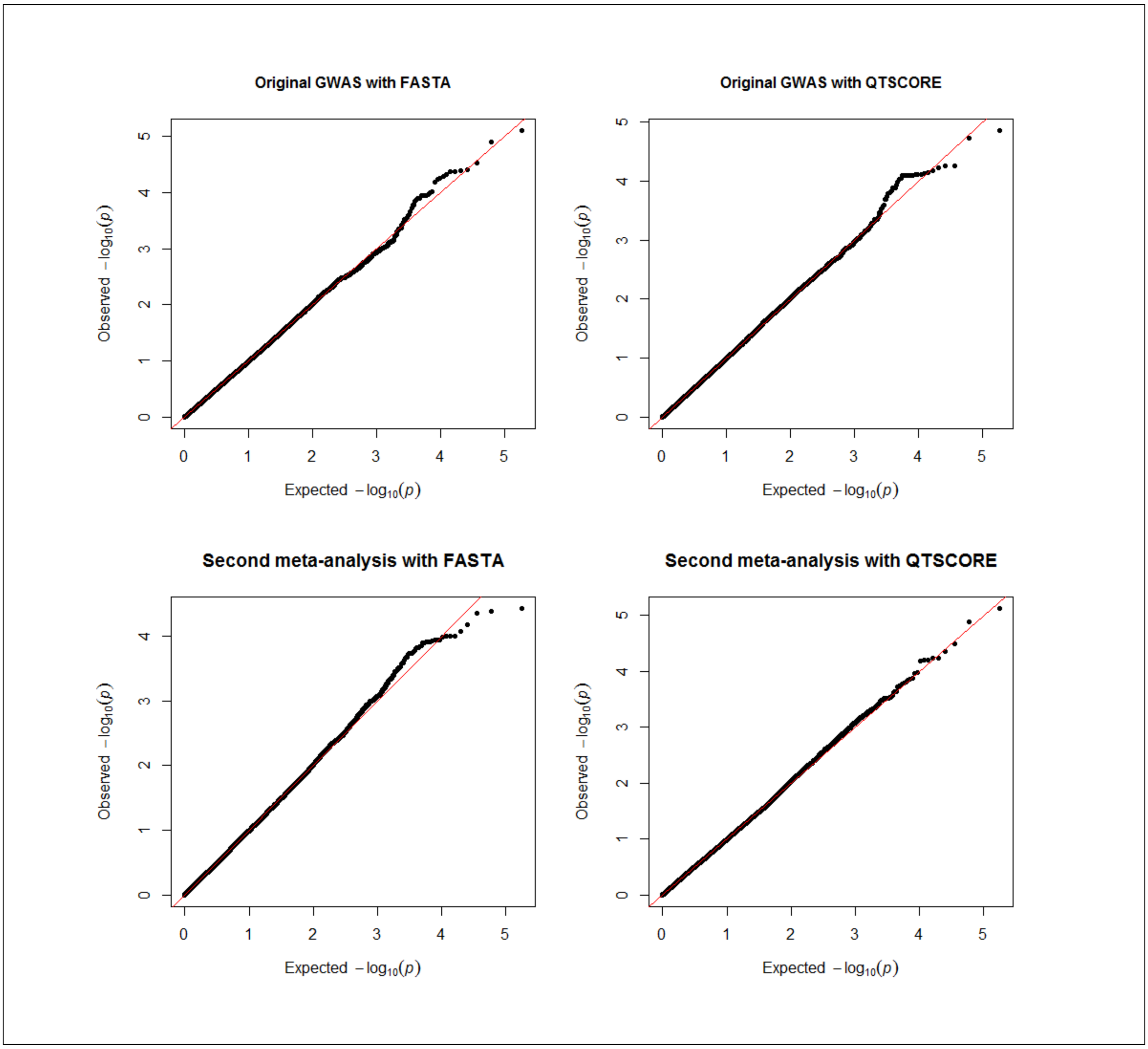

Q-Q plots with different analysis methods: FASTA vs. meta-analysis with FASTA, QTSCORE vs. meta-analysis with QTSCORE.

We assume that the loss of power in the second meta-analysis was due to inability of the analysis method to handle the increased variation within the cases after inclusion of the less extreme hip dysplasia phenotypes (C/C, C/D, and D/C), as the first step for FASTA indicated bad fit.

The deflation of the lambda for QTSCORE, when used with the environmental residuals, as in the original GWAS and first meta-analysis, is due to the conservative nature of the test and is common when studying polygenic traits in small populations with inadequate power.
