# Supplementary figures and images for: Novel protective and risk loci in hip dysplasia in German Shepherds
Source: PLoS Genet. 2019 Jul 19;15(7):e1008197. doi: 10.1371/journal.pgen.1008197 (PMC6668854; doi:10.1371/journal.pgen.1008197)

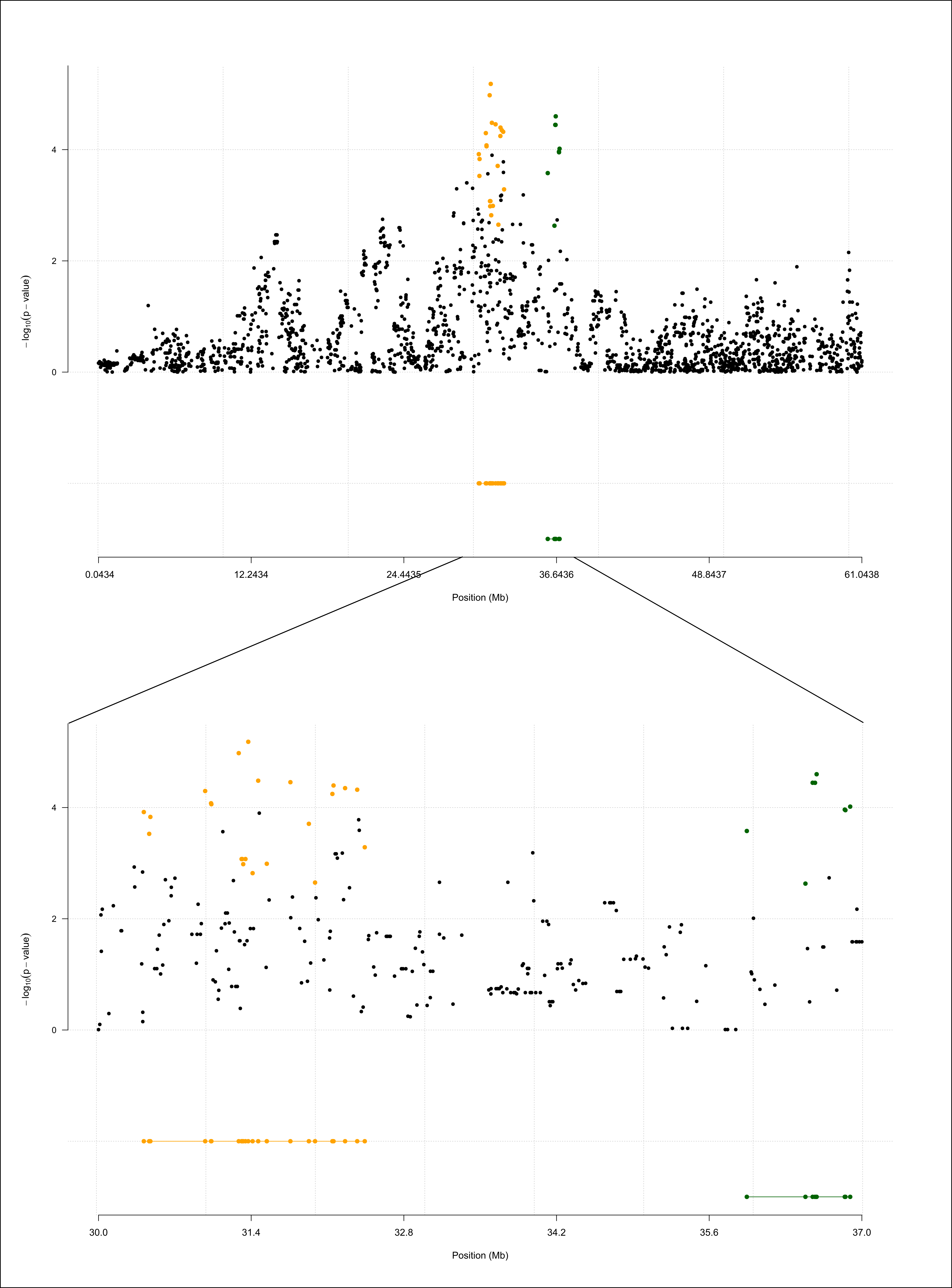

Supplement: S2 Fig — Yellow = first locus near NOG. Green = second locus near LHX1. (TIFF) [file pgen.1008197.s002.tiff]

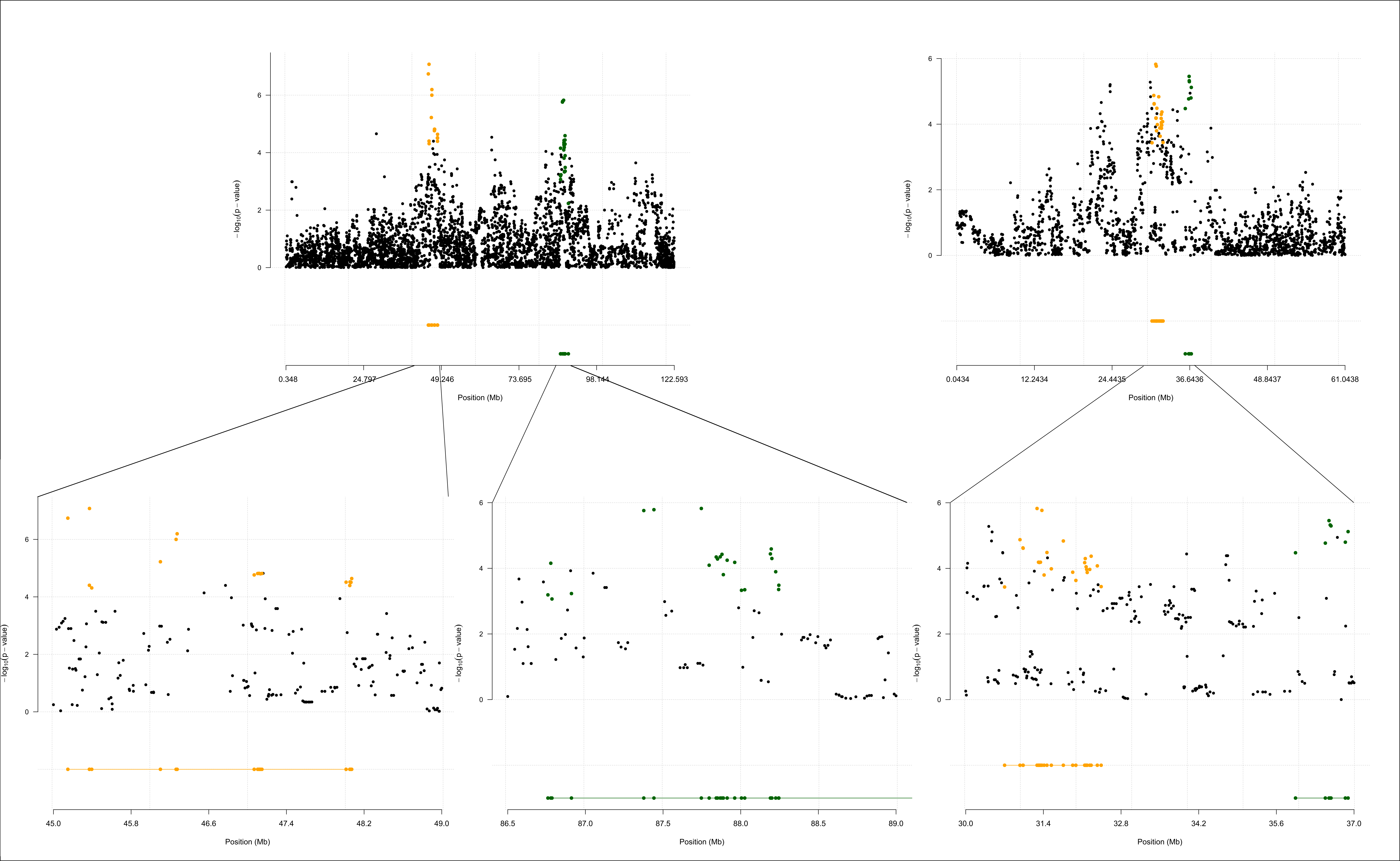

Supplement: S3 Fig — Left panel: yellow = first locus near NOX3 and ARID1B, green = second locus near MAMDC2 and PTAR1. Right panel: Yellow = first locus near NOG. Green = second locus near LHX1. (TIFF) [file pgen.1008197.s003.tiff]

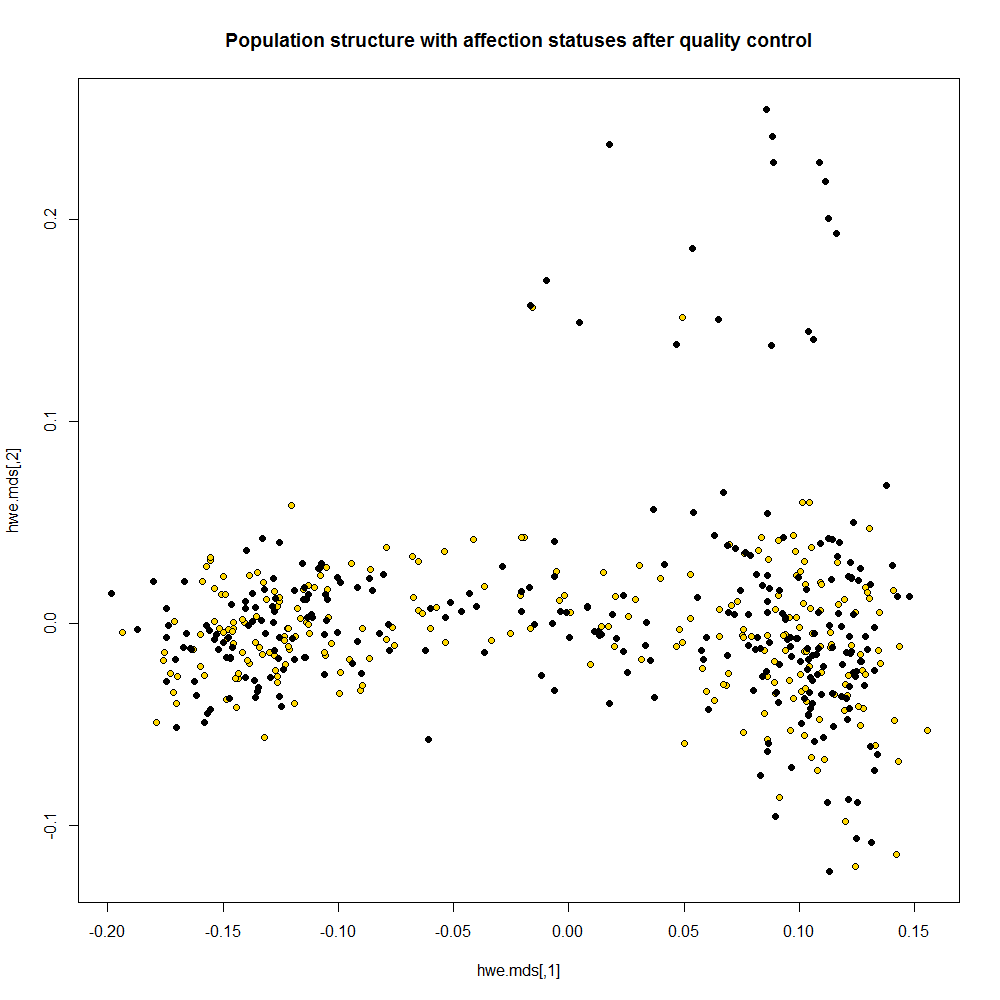

Supplement: S10 Fig — Cases (representing either mild, moderate or severe hip dysplasia) are marked with yellow and controls are marked with black. This figure includes all of the 525 dogs used in the association analyses. The right-hand cluster in this figure consist of working line dogs and the left-hand cluster of show line dogs; mixed line animals are between these two main groups (dogs whose ancestors have both show and working line animals). The separate cluster above the working line cluster consists of a group of closely related dogs (full- and half-siblings and their common female ancestor), and a dog sharing multiple different common ancestors with this family-group. (TIFF) [file pgen.1008197.s010.tiff]

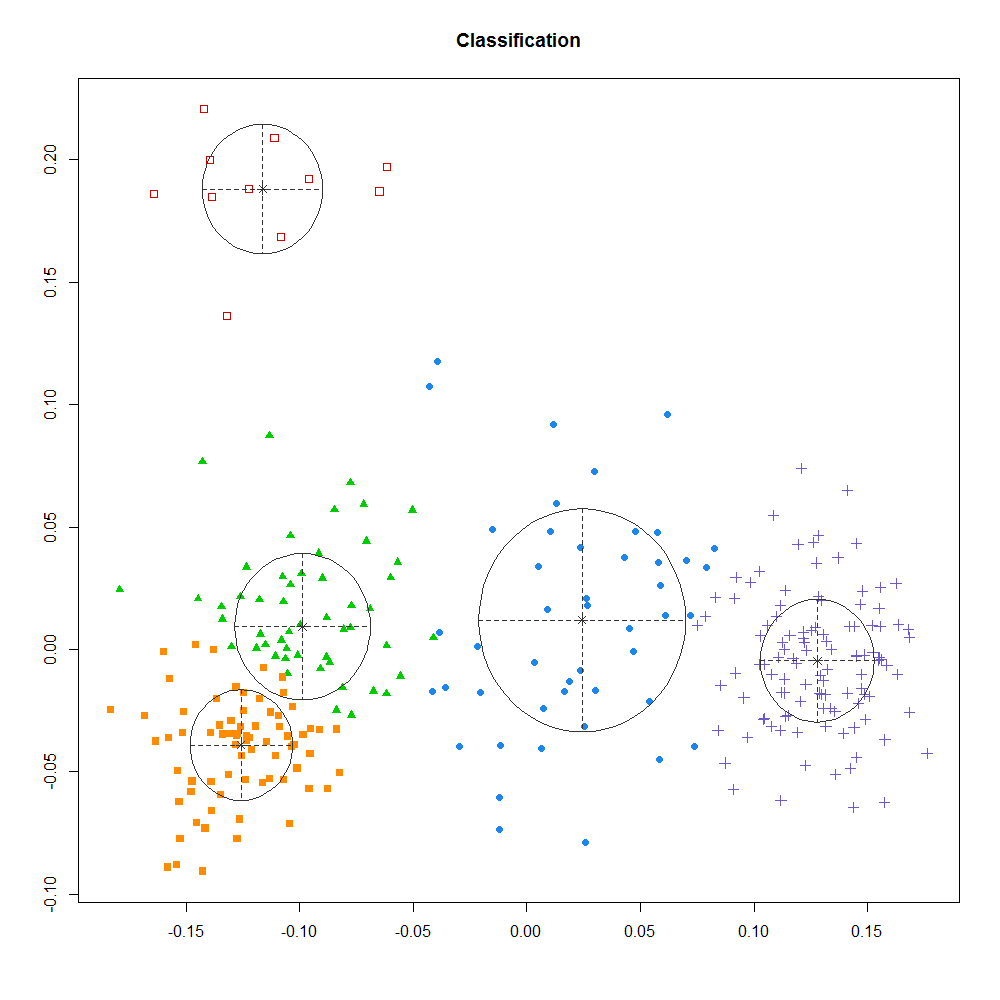

Supplement: S11 Fig — Best model by mclust: spherical, varying volume with 5 components. (TIFF) [file pgen.1008197.s011.tiff]

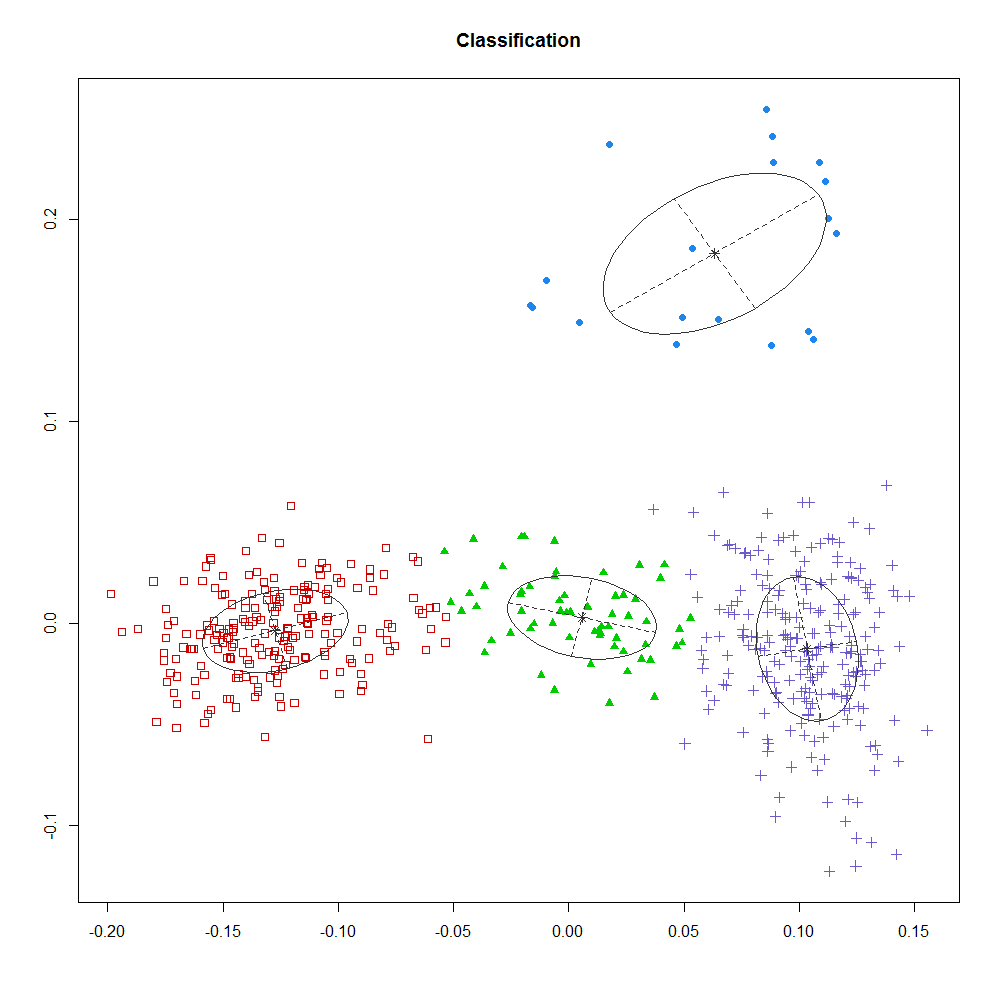

Supplement: S12 Fig — Best model by mclust: ellipsoidal, equal shape with 4 components. (TIFF) [file pgen.1008197.s012.tiff]
