# Supplementary material for: Novel protective and risk loci in hip dysplasia in German Shepherds
Source: PLoS Genet. 2019 Jul 19;15(7):e1008197. doi: 10.1371/journal.pgen.1008197 (PMC6668854; doi:10.1371/journal.pgen.1008197)
Supplement: S4 Fig — The M2 test variable for each SNP was calculated from a permutated cohort in S4 Table. The distribution of the maximum value of M2 from 10000 permutations is indicated. Vertical lines indicate the mean value and its upper and lower bound with 95% confidence interval. The dotted line indicates the value of M2 = 24.10 corresponding to a Bonferroni-adjusted p-value of 0.05. Bandwidth = 0.5. (PDF) [file pgen.1008197.s004.pdf]

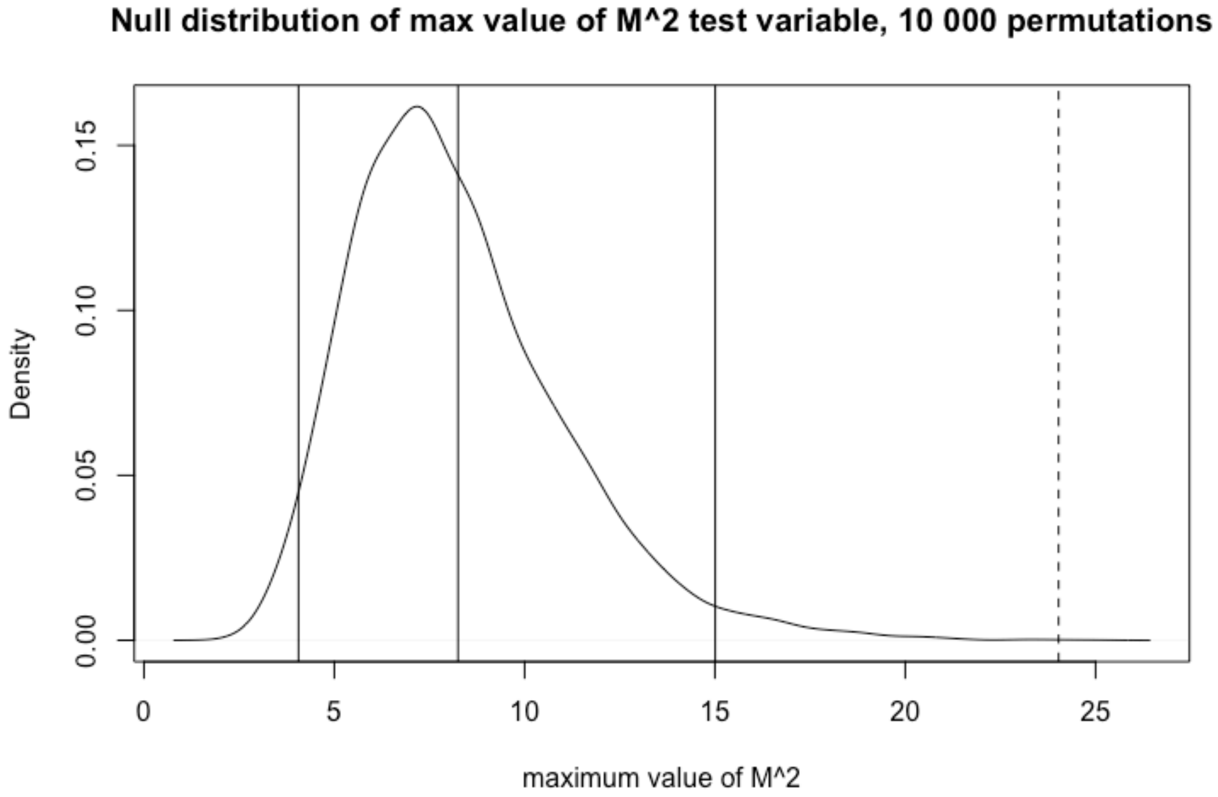

The null distribution of the maximum value of the M^2 test variable. The M^2 test variable for each SNP was calculated from a permutated cohort in S4 Table. The distribution of the maximum value of M^2 from 10000 permutations is indicated. Vertical lines indicate the mean value and its upper and lower bound with 95% confidence interval. The dotted line indicates the value of M^2=24.10 corresponding to a Bonferroni-adjusted p-value of 0.05. Bandwidth = 0.5
