# Supplementary material for: Novel protective and risk loci in hip dysplasia in German Shepherds
Source: PLoS Genet. 2019 Jul 19;15(7):e1008197. doi: 10.1371/journal.pgen.1008197 (PMC6668854; doi:10.1371/journal.pgen.1008197)
Supplement: S5 Fig — Bandwith = 700 kb. The positions of bases on chromosome 9 are indicated on the x-axis. The green vertical lines indicate the positions of the 28 SNPs that associate with CHD. (See the bolded lines in S5 Table) The black vertical line indicates the position of the deletion upstream NOG at 31453837–31453860. (PDF) [file pgen.1008197.s005.pdf]

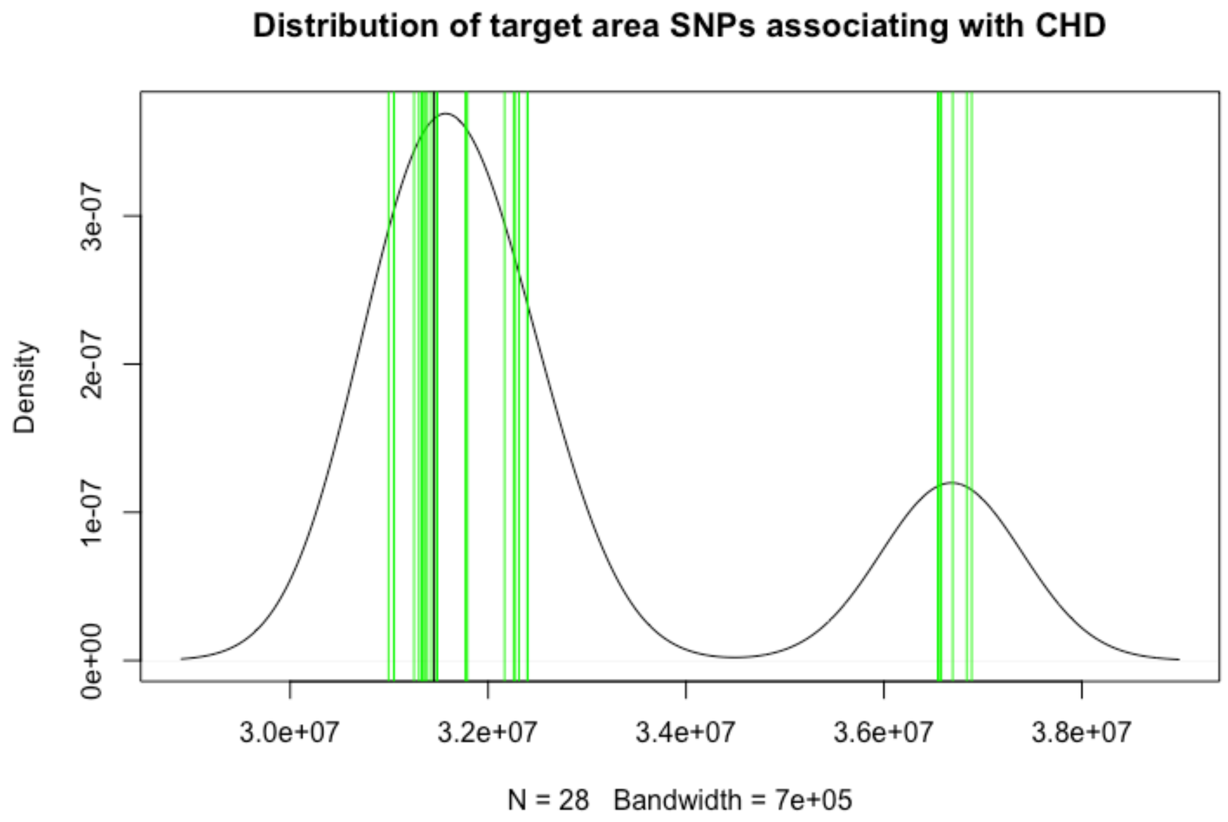

Densitogram of the statistically significant SNPs along the resequenced target area. Bandwith = 700 kb. The positions of bases along chromosome 9 are indicated on the x-axis. The green vertical lines indicate the positions of the 28 SNPs that associate with CHD. (See the bolded lines in S5 Table.) The black vertical line indicates the position of the deletion upstream *NOG* at 31453837-31453860.
