# Supplementary material for: Novel protective and risk loci in hip dysplasia in German Shepherds
Source: PLoS Genet. 2019 Jul 19;15(7):e1008197. doi: 10.1371/journal.pgen.1008197 (PMC6668854; doi:10.1371/journal.pgen.1008197)
Supplement: S7 Fig — The position for the last nucleotide in the alignment is indicated for construct A and the CanFam3.1 chromosome 9 reference (NC_006591.3). Differences among any of the sequences are indicated by asterisks (*). Differences between construct A and CanFam3.1 are painted yellow. (PDF) [file pgen.1008197.s007.pdf]

```
construct A     ctcaggaagttcgtttacaccgagggcctggaaagcttggagagcacagacccgagcagt
Dog2-1          ctcaggaagttcgtttacaccgagggcctggaaagcttggagagcacagacccgagcagt
Dog2-2          ------------------------------------------------------------
Dog1            ctcaggaagttcgtttacaccgagggcctggaaagcttggagagcacagacccgagcagt
Dog5-1          ctcaggaagttcgtttacaccgagggcctggaaagcttggagagcacagacccgagcagt
Dog5-2          ------------------------------------------------------------
NC_006591.3     ctcaggaagttcgtttacaccgagggcctggaaagcttggagagcacagacccgagcagt

construct A     ccaggcccccgca--gcgcgcgcgcacgcacacactatgacacggacacgcgagcagaag
Dog2-1          ccaggcccccgca--gcgcgcgcgcacgcacacactatgacacggacacgcgagcagaag
Dog2-2          ------------------------------------------------------------
Dog1            ccaggcccccgca--gcgcgcgcgcacgcacacactatgacacggacacgcgagcagaag
Dog5-1          ccaggcccccgcagcgcgcgcgcgcacgcacacactatgacacggacacgcgagcagaag
Dog5-2          ------------------------------------------------------------
NC_006591.3     ccaggcccccgcagcgcgcgcgcgcacgcacacactatgacacggacacgcgagcagaag
                             **

construct A     cggagctgggccgggcggaccgcaggcgaacccacctcgcggggccgcccccgccagcgc
Dog2-1          cggagctgggccgggcggaccgcaggcgaacccacctcacgggg----------------
Dog2-2          ------------------------ggcgagcccgcctcgcggggccgcccccgccagcgc
Dog1            cggagctgggccgggcggaccgcaggcgaacccacctcgcggggccgcccccgccagcgc
Dog5-1          cggagctgggccgggcggaccgcaggcgaacccacctcgcggggccgcccccgccagcgc
Dog5-2          ------------------------------------------------------------
NC_006591.3     cggagctgggccgggcggaccgcaggcgaacccacctcgcggggccgcccccgccagcgc
                                             *   *    *

construct A     caggtgcaggactcgccgggcgccagcagagggcgcgccgcccccagggagccgagcagc
Dog2-1          ------------------------------------------------------------
Dog2-2          caggtgcaggactcgccgggcgccagcagagggcgcgccgcccccagggagccgagcagc
Dog1            caggtgcaggactcgccgggcgccagcagagggcgcgccgcccccagggagccgagcagc
Dog5-1          caggtgcaggactcgccgggcgccagcagagggcgcgccgcccccagggagccgagcagc
Dog5-2          ------------------------------------------------------------
NC_006591.3     caggtgcaggactcgccgggcgccagcagagggcgcgccgcccccagggagccgagcagc

construct A     ggcggcgcggaacccggccggctaggggagccgcaggcgagccggacgcgcccctgcagc
Dog2-1          ------------------------------------------------------------
Dog2-2          ggcggcgcggaacccggccggctaggggagccgcaggcgagccggacgcgcccctgcagc
Dog1            ggcggcgcggaacccggccggctaggggagccgcaggcgagccggacgcgcccctgcagc
Dog5-1          ggcggcgcggaacccggccggctaggggagccgcaggcgagccggacgcgcccctgcagc
Dog5-2          ------------------------------------------------------------
NC_006591.3     ggcggcgcggaacccggccggctaggggagccgcatgcgagccggacgcgcccctgcagc
                                                   *

construct A     tcggccactcagggtgcaggacccggccaaggaggaggaggaggaggaggaggaggagga
Dog2-1          ------------------------------------------------------------
Dog2-2          tcggccactcagggtgcaggacccggccaaggaggaggaggaggaggaggaggaggagga
Dog1            tcggccactcagggtgcaggacccggccaaggaggaggaggaggaggtggaggaggagga
Dog5-1          tcggccactcagggtgcaggacccggccaaggaggaggaggaggaggaggaggaggagga
Dog5-2          ------------------------------------------------------------
NC_006591.3     tcggccactcagggtgcaggacccggccaaggaggaggaggaggaggagaaggaggagga
                                                               *

construct A     ggagg---aggagggcgaggagcggaccccctcgtgggcccgagtaacagacgggcagg
Dog2-1          ------------------------------------------------------------
Dog2-2          ggagg---aggagggcgaggagcggaccccctcgtgggcccgagtaacagacgggcagg
Dog1            ggaggaggaggagggcgaggagcggacac-------------------------------
Dog5-1          ggagg-------------------------------------------------------
Dog5-2          ------------------------------------------------------------
NC_006591.3     ggaggaggaggagggcgaggagcggaccccctcgtgggcccgagtaacagacgggcagg
                     ***                   *
```

```
construct A     agtgcttcctaggaattacatcacgctgcgcaacgctgcggctctcggagtcctgctcaa
Dog2-1          ------------------------------------------------------------
Dog2-2          agtgcttcctaggaattacatcacgctgcgcaacgctgcggctctcggagtcctgctcaa
Dog1            ------------------------------------------------------------
Dog5-1          ------------------------------------------------------------
Dog5-2          -------------------------------------------------gtcctgctcaa
NC_006591.3     agtgcttcctaggaattacatcacgctgcgcaacgctgcggctctcggagtcctgctcaa

construct A     agtgtgtgtgtgtgtgtgtgtgtgtgtgtgtgt--------gtgtgtgtgtgtacgcgct
Dog2-1          ------------------------------------------------------------
Dog2-2          agtgtgtgtgtgtgtgtgtgtgtgtgtgtgtgt--------gtgtgtgtgtgtacgcgct
Dog1            ------------------------------------------------------------
Dog5-1          ------------------------------------------------------------
Dog5-2          agtgtgtgtgtgtgtgtgtgtgtgtgtgtgtgt--------gtgtgtgtgtgtacgcgct
NC_006591.3     agtgtgtgtgtgtgtgtgtgtgtgtgtgtgtgtgtgtgtgagtgtgtgtgtgtacgcgct
                                                 ********

construct A     ggggggaggggactcagagccagctccggagagaggaaggcga 570
Dog2-1          -------------------------------------------
Dog2-2          ggggggaggggactcagagccagctccggagagaggaaggcga
Dog1            -------------------------------------------
Dog5-1          -------------------------------------------
Dog5-2          ggggggaggggactcagagccagctccggagagaggaaggcga
NC_006591.3     ggggggaggggactcagagccagctccggagagaggaaggcga 31454090
```
