# Supplementary material for: Novel protective and risk loci in hip dysplasia in German Shepherds
Source: PLoS Genet. 2019 Jul 19;15(7):e1008197. doi: 10.1371/journal.pgen.1008197 (PMC6668854; doi:10.1371/journal.pgen.1008197)
Supplement: S8 Fig — (PDF) [file pgen.1008197.s008.pdf]

```
Dog6                1 aaccggtgccaacgtgcgcggacgccgccgccgccgccgccgctggagtccgcctggcag
NC_006591.3 31454789 aaccggtgccaacgtgcgcggacgccgccgccgccgccgccgctggagtccgccgggcag

Dog6               61 agccggccgcggagccccgagcaggcggagggacgcgccccggacccgctgcgccagccg
NC_006591.3 31454849 agccggccgcggagccccgagcaggcggagggacgcgccccggacccgctgcgccagccg

Dog6              121 cgctgccccgggcggcgggacgaccgcggcgagcggaggaggaggaggaggggagggcgg
NC_006591.3 31454909 cgctgccccgggcggcggcacga-agcggcgagcggacgacgaac---------------

Dog6              181 ctcggccccgcgccccgcgcccgcgcccgcgccccgcgccccgcgccccgcgccccgcgc

Dog6              241 cgccgcccgggagcagcgaggagccggcgccccccgcgcccgccggcgccctggagtccc
AB544074.1          1 ----------------------gccggcgccccccgcgcccgccggcgccctggagtccc

Dog6              301 cgcggacgccccgccgcggccgccggccccagtagacccggcggagcagccgcggccgac
AB544074.1         39 cgcggacgccccgccgcggccgccggccccagtagacccggcggagcagccgcggccgac

Dog6              361 ccgtgcgccgcctcccgcccgccccggtgggagccggcgccgcgcgaagggctctcccgg
AB544074.1         99 ccgtgcgccgcctcccgcccgccccggtgggagccggcgccgcgcgaagggctctcccgg

Dog6              421 cgcctcatgctgccggccctgcgcccgcccagccgcgggtgagccgcctccggagggccg
AB544074.1        159 cgcctcatgctgccggccctgcgcccgcccagccgcgggtgagccgcctccggagggccg

Dog6              481 gggagtgctctcctcgggggacgcggacgcggcggcagccccgggcgcgccggaggcatg
AB544074.1        219 gggagtgctctcctcgggggacgcggacgcggcggcagccccgggcgcgccggaggcatg

Dog6              541 gagcgctgccccagcctgggggtcaccctctacgccctggtggtggtcctggggctgcgg
NC_006591.3 31455387 -------------------------------------------tggtcctggggctgcgg
AB544074.1        279 gagcgctgccccagcctgggggtcaccctctacgccctggtggtggtcctggggctgcgg

Dog6              601 gcggcaccggccggcggccagcactacctccacatccgcccggcccccagcgacaacctg
NC_006591.3 31455404 gcggcaccggccggcggccagcactacctccacatccgcccggcccccagcgacaacctg
AB544074.1        339 gcggcaccggccggcggccagcactacctccacatccgcccggcccccagcgacaacctg

Dog6              661 cccctggtggacctcatcgagcacccggaccctatctttgaccccaaggagaaggatctg
NC_006591.3 31455464 cccctggtggacctcatcgagcacccggaccctatctttgaccccaaggagaaggatctg
AB544074.1        399 cccctggtggacctcatcgagcacccggaccctatctttgaccccaaggagaaggatctg

Dog6              721 aacgagacgctgctgcgctcgctgctcgggggccactacgacccgggcttcatggccacc
NC_006591.3 31455724 aacgagacgctgctgcgctcgctgctcgggggccactacgacccgggcttcatggccacc
AB544074.1        459 aacgagacgctgctgcgctcgctgctcgggggccactacgacccgggcttcatggccacc

Dog6              781 tcgccccccgaggagcggcccggcgggggcgggggcgcgggcgcgggcgcgggcgc-ggc
NC_006591.3 31455784 tcgccccccgaggagcggcccggcgggggcgggggcgcgggcgcgggcgcgggcgcgggc
AB544074.1        519 tcgccccccgaggagcggcccggcgggggcgggggcgcgggcgcgggcgcgggcgc-ggc

Dog6              840 cgggggcgccgaggacctggccgagctggaccagctgctgcggcagcggccgtcgggggc
NC_006591.3 31455844 cgggggcgccgaggacctggccgagctggaccagctgctgcggcagcggccgtcgggggc
AB544074.1        578 cgggggcgccgaggacctggccgagctggaccagctgctgcggcagcggccgtcgggggc

Dog6              900 catgccgagcgagatcaaagggctggagttctccgagggcttggccccgggcaagaagca
NC_006591.3 31455904 catgccgagcgagatcaaagggctggagttctccgagggcttggccccgggcaagaagca
AB544074.1        638 catgccgagcgagatcaaagggctggagttctccgagggcttggccccgggcaagaagca

Dog6              960 gc 961
NC_006591.3 31455764 gc 31455765
AB544074.1        698 gc 699
```
