# Supplementary material for: Novel protective and risk loci in hip dysplasia in German Shepherds
Source: PLoS Genet. 2019 Jul 19;15(7):e1008197. doi: 10.1371/journal.pgen.1008197 (PMC6668854; doi:10.1371/journal.pgen.1008197)
Supplement: S9 Fig — For clarity, the artificial numbering of the sequence from Dog6 corresponds the location of 434 bp gap on the chromosome 9 sequence (NC_006591.3). (PDF) [file pgen.1008197.s009.pdf]

```
Dog6          31454953 aggaggggagggcggctcggccccgcgccccgcgcccgcgcccgcgccccgcgccccgcg
GRCh38 chr17 56593860 aggaggggagagcggctcgtccacgcgccctgcg--------------------------
                      **********.******** ** *******.***

Dog6                  ccccgcgccccgcgccgccgcccgggagcagcgaggagccggcgccccccgcg-cccgcc
GRCh38 chr17          --------ccgccgccggcccgggaaggcagcgaggagccggcgcctcccgcgccccgcg
                              **  ***** * *  *...*******************.****** *****

Dog6                  ggcgccctggagtccccgcggacgccccgccgcggccgccggccccagtagacccggcgg
GRCh38 chr17          gtcgccctggagtaatttcggatgcccagccgcggccgccttccccagtagacccgggag
                      * ***********  .. ****.**** ************  *************** .*

Dog6                  agcagccgcggccgacccgtgcgccgcctcccgcccgccccggtgggagccggcgccgcg
GRCh38 chr17          aggagttgcggccaacttgtg---tgcctttcttccgccccggtgggagccggcgctgcg
                      ** **..******.**..***   .****..* .**********************.***

Dog6                  cgaagggctctcccggcgcctcatgctgccggccctgcgcccgcccagccgcgggtgagc
GRCh38 chr17          cgaagggctctcccggcggctcatgctgccggccctgcgcctgcccagcctcgggtgagc
                      ****************** **********************.******** *********

Dog6                  cgcctccggagggccgggga-------------------------gtgctctcctcgggg
GRCh38 chr17          cgcctccggagagacggggagcgcggcggcgccgcgggctcggcgtgctctcctccggg
                      ***********.* *****.                         *********** ***

Dog6                  gacgc-ggacgcggcggcagccccgg--gcgcgccggaggcatggagcgctgccccagcc
GRCh38 chr17          gacgcgggacgaagcagcagccccgggcgcgccagaggcatggagcgctgccccagcc
                      ***** ***** .**.**********  *******.************************

Dog6                  tgggggtcaccctctacgccctggtgg 31455386
GRCh38 chr17          taggggtcaccctctacgccctggtgg 56594269
                      *.*************************
```
