# Supplementary material for: Novel protective and risk loci in hip dysplasia in German Shepherds
Source: PLoS Genet. 2019 Jul 19;15(7):e1008197. doi: 10.1371/journal.pgen.1008197 (PMC6668854; doi:10.1371/journal.pgen.1008197)
Supplement: S7 Table — (PDF) [file pgen.1008197.s019.pdf]

Name	Sequence
Construct A	CTCAGGAAGTTCGTTTACACCGAGGGCCTGGAAAGCTTGGAGAGC<br>ACAGACCCGAGCAGTCCAGGCCCCCGCAGCGCGCGCACGCACAC<br>ACTATGACACGGACACGCGAGCAGAAGCGGAGCTGGGCCGGGCG<br>GACCGCAGGCGAACCCACCTCGCGGGGCCGCCCCCGCCAGCGCCA<br>GGTGCAGGACTCGCCGGGCGCCAGCAGAGGGCGCGCCGCCCCCAG<br>GGAGCCGAGCAGCGGCGGCGCGGAACCCGGCCGGCTAGGGGAGC<br>CGCAGGCGAGCCGGACGCGCCCCTGCAGCTCGGCCACTCAGGGTG<br>CAGGACCCGGCCAAGGAGGAGGAGGAGGAGGAGGAGGAGG<br>AGGAGGAGGAGGGCGAGGAGCGGACCCCCCTCGTGGGCCCGAGT<br>AACAGACGGGCAGGAGTGCTTCCTAGGAATTACATCACGCTGCGCA<br>ACGCTGCGGCTCTCGGAGTCCTGCTCAAAGTGTGTGTGTGTGTGT<br>GTGTGTGTGTGTGTGTGTGTGTGTGTGTGTACGCGCTGGGGGGAGG<br>GGACTCAGAGCCAGCTCCGGAGAGAGGAAGGCGA
Construct B	CTCAGGAAGTTCGTTTACACCGAGGGCCTGGAAAGCTTGGAGAGC<br>ACAGACCCGAGCAGTCCAGGCCCCCGCAGCGCGCGCACGCACAC<br>ACTATGACACGGACACGCGAGCAGAAGCGGAGCTGGGCCGGGCG<br>GACCGCAGGCGAACCCACCTCGCGGGGCCGCCCCCGCCAGCGCCA<br>GGTGCAGGACTCGCCGGGCGCCAGCAGAGGGCGCGCCGCCCCCAG<br>GGAGCCGAGCAGCGGCGGCGCGGAACCCGGCCGGCTAGGGGAGC<br>CGCAGGCGAGCCGGACGCGCCCCTGCAGCTCGGCCACTCAGGGTG<br>CAGGACCCGGCCAAGGAGGAGGAGGAGGGCGAGGAGCGGA<br>CCCCCCTCGTGGGCCCGAGTAACAGACGGGCAGGAGTGCTTCCTA<br>GGAATTACATCACGCTGCGCAACGCTGCGGCTCTCGGAGTCCTGCT<br>CAAAGTGTGTGTGTGTGTGTGTGTGTGTGTGTGTGTGTGTGTGTGTG<br>TGTACGCGCTGGGGGGAGGGGACTCAGAGCCAGCTCCGGAGAGA<br>GGAAGGCGA
Construct C	CTCAGGAAGTTCGTTTACACCGAGGGCCTGGAAAGCTTGGAGAGC<br>ACAGACCCGAGCAGTCCAGGCCCCCGCAGCGCGCGCACGCACAC<br>ACTATGACACGGACACGCGAGCAGAAGCGGAGCTGGGCCGGGCG<br>GACCGCAGGCGAACCCACCTCGCGGGGCCGCCCCCGCCAGCGCCA<br>GGTGCAGGACTCGCCGGGCGCCAGCAGAGGGCGCGCCGCCCCCAG<br>GGAGCCGAGCAGCGGCGGCGCGGAACCCGGCCGGCTAGGGGAGC<br>CGCAGGCGAGCCGGACGCGCCCCTGCAGCTCGGCCACTCAGGGTG<br>CAGGACCCGGCCAAGGAGGAGGAGGAGGAGGGCGAGGAGC<br>GGACCCCCCTCGTGGGCCCGAGTAACAGACGGGCAGGAGTGCTTC<br>CTAGGAATTACATCACGCTGCGCAACGCTGCGGCTCTCGGAGTCCT<br>GCTCAAAGTGTGTGTGTGTGTGTGTGTGTGTGTGTGTGTGTGTGTGT<br>GTGTGTACGCGCTGGGGGGAGGGGACTCAGAGCCAGCTCCGGAG<br>AGAGGAAGGCGA

Construct sequences A to C for Dual luciferase reporter assay design.
