# Supplementary material for: Novel protective and risk loci in hip dysplasia in German Shepherds
Source: PLoS Genet. 2019 Jul 19;15(7):e1008197. doi: 10.1371/journal.pgen.1008197 (PMC6668854; doi:10.1371/journal.pgen.1008197)
Supplement: S9 Table — The human core JASPAR2018 database was queried with the alignment of dog and human sequences in S9 Fig. The matrix ID’s for which the bonferroni-corrected P-value for any binding site is less than 0.05, are presented together with the corresponding transcription factors. (PDF) [file pgen.1008197.s021.pdf]

TOP hits for transcription factor with predicted binding sites in the dog genomic sequence in S9 Fig.

adjusted P (minimum)	ID	name	family
0.00211691856384277	MA0131.2	HINFP	Factors with multiple dispersed zinc fingers
0.00358247756958008	MA0471.1	E2F6	E2F-related factors
0.00879335403442383	MA0470.1	E2F4	E2F-related factors
0.0169353485107422	MA0814.1	TFAP2C(var.2)	AP-2
0.0192151069641113	MA0812.1	TFAP2B(var.2)	AP-2
0.0276827812194824	MA0003.3	TFAP2A	AP-2

For each matrix ID the minimum of the Bonferroni corrected (N=1316) p values is presented. For the detailed descriptions of predicted binding sites for each matrix ID, see S8 Table.
