# Supplementary material for: Novel protective and risk loci in hip dysplasia in German Shepherds
Source: PLoS Genet. 2019 Jul 19;15(7):e1008197. doi: 10.1371/journal.pgen.1008197 (PMC6668854; doi:10.1371/journal.pgen.1008197)
Supplement: S11 Table — (PDF) [file pgen.1008197.s023.pdf]

	Sequence (5' > 3')	Strand	Length	Tm (Primer-BLAST / ThermoFisher Scientific Tm calculator)	GC %
Forward primer	GGCCTGGAAAGCTTGGAGAG	Plus	20	60.68 / 67.6	60
Reverse primer	CACTCCTGCCCGTCTGTTAC	Minus	20	60.39 / 65.1	60
Product length	400				

Primer details for PCR and fragment analysis.
