# Supplementary material for: Novel protective and risk loci in hip dysplasia in German Shepherds
Source: PLoS Genet. 2019 Jul 19;15(7):e1008197. doi: 10.1371/journal.pgen.1008197 (PMC6668854; doi:10.1371/journal.pgen.1008197)
Supplement: S6 Fig — The sequences are from Ensemble, version 94. The stars indicate the insert in Fig 4 spanning over GRCh38 17: 56592775–56592815. This region corresponds to the 5’ NOG core promoter (ENSR00000096009 in Ensembl v.94 [33]) in the human chromosome 17: 56,592,600–56,594,601 (GRCh38.p12). The prealigned sequences from 26 eutherian mammals with the human NOG core promoter can be found at: http://oct2018.archive.ensembl.org/Homo_sapiens/Share/cea4ebce4ec4fa397e367c647cf86f8f?redirect=no;mobileredirect=no. (PDF) [file pgen.1008197.s006.pdf]

```
                                               **********************************************************************
Human                            17: 56592749 GAGGCTAGGCCACTCAGGGTGCAGGACCCGGCCAAGGAGGAGGAGGATG-----------------------------GTGAGGAGCGGAACCCCCTCGTGGGCCCGAGGGGCAGACAGACAGGA
Bonobo                           17: 55531092 GAGGCTAGGCCACTCAGGGTGCAGGACCCGGCCAAGGAGGAGGAGGATG-----------------------------GTGAGGAGCGGAACCCCCTCGTGGGCCCGAGGGGCAGACAGACAGGA
Chimpanzee                       17: 55837523 GAGGCTAGGCCACTCAGGGTGCAGGACCCGGCCAAGGAGGAGGAGGATG-----------------------------GTGAGGAGCGGAACCCCCTCGTGGGCCCGAGGGGCAGACAGACAGGA
Gorilla                           5: 26805235 GAGGCTAGGCCACTCAGGGTGCAGGACCCAGCCAAGGAGGAGGAGGATG-----------------------------GTGAGGAGCGGAACCCCCTCGTGGGCCCGAGGGGCAGACAGACAGGA
Orangutan                        17: 35638525 GAGGCTAGGCCACTCAGGGTGCAGGACCCGGCCAAGGAGGAGGAGGATG-----------------------------GTGAGGAGCTGAACCCCCTCGTGGGCCCGAGGAGCAGACAGACAGGA
Gibbon                           14:  7107375 GAGGCTAGGCCACTCTGGGTGCAGGACCCGGCCAAGGAGGAGGAGGATG-----------------------------GTGAGGAGCGGAACCTCCTCGTGGGCCCGAGGGGCAGACAGACAGGA
Vervet-AGM                       16: 36819658 GAGGCTAGGCCACTCAGGGTGCAGGACCCGGCCAAGGAGGAGGAAGATG-----------------------------GTGAGGAGCGGACCCCCCTCGTGGGCCCGAAGGACAGACAGGCAGGA
Crab-eating macaque              16: 42070250 GAGGCTAGGCCACTCAGGGTGCAGGACCCGGCCAAGGAAGAGGAAGATG-----------------------------GTGAGGAGCGGACCCCCCTCGTGGGCCCGAAGGACAGACAGGCAGGA
Macaque                          16: 33086049 GAGACTAGGCCACTCAGGGTGCAGGACCCGGCCAAGGAAGAGGAAGATG-----------------------------GTGAGGAGCGGACCCCCCTCGTGGGCCCGAAGGACAGACAGGCAGGA
Olive baboon                     16: 37660602 GAGGCTAGGCCACTCAGGGTGCAGGACCCGGCCAAGGAAGAGGAAGATG-----------------------------GTGAGGAGCGGACCCCCCTCGTGGGCCCGAAGGACAGACAGGCAGGA
Marmoset          NTIC01034389.1:103271651 GAGGCTAGGCCAGTCAGGGTGCAGAACCCGGCCAAGGAGGAGGAGGATG-----------------------------GTGAGGAGCGGACTCCCCTCGTGGGCCCGAGGGCCAGACAGGCAGGA
Mouse Lemur                      16: 19875235 GCGGCTCGGCCACTCAGGGTGCAGGACCCGGCCAAGGAGGAGGAGGAGG-----------------------------GTGAGGAGCGGA-CCCCCTCGTGGGCCTGGGGGGCAGACGGGCAGGA
Rabbit                           19: 31417997 GCGGCTGGGCCACTCAGGGTGCAGGACCCGGCCAAGGAGGAGGAGGAAGAGG-----------------------AGGGTGAGGAGCGAACCCCCATCGTGGGCCCGGGGGTCAGACGGGCAGGA
Dog                               9: 31453810 GCAGCTCGGCCACTCAGGGTGCAGGACCCGGCCAAGGAGGAGGAGGAGGAGGAGAAGGAGGAGGAGGAGGAGGAGGGCGAGGAGCGGACCCCCCTCGTGGGCCCGAGTAACAGACGGGCAGGA
Cat                              E1: 32065737 GCAGCTCGGCCACTCAGGGTGCAGGACCCGGCCAAGGAGGAGGAGG--------------------------------GTGAAGAGCGGACCCCCCTCGTGGGCCCGAGTAACAGACGGGCCGGA
                                               *********************************************************************
```

Manually adjusted multiple alignment of the triplet-repeat region upstream of *NOG* from 15 species representing primates, lagomorphs, canidae and felidae. The sequences are from Ensemble, version 94. The stars indicate the insert in Fig 4 spanning over GRCh38 17: 56592775-56592815.

This region corresponds to the 5' *NOG* core promoter (ENSR00000096009 in Ensembl v.94, Derbino et al. (2018) Nucl. Acid Res. 46:D754-D761) in the human chromosome 17: 56,592,600-56,594,601 (GRCh38.p12). The prealigned sequences from 26 eutherian mammals (*) with the human *NOG* core promoter can be found at http://oct2018.archive.ensembl.org/Homo_sapiens/Share/cea4ebce4ec4fa397e367c647cf86f8f?redirect=no;mobileredirect=no.

(*)

Human	chromosome:GRCh38:17:56592600:56594601:1
Bonobo	chromosome:panpan1.1:17:55530943:55532771:1
Chimpanzee	chromosome:Pan_tro_3.0:17:55837374:55839379:1
Gorilla	chromosome:gorGor4:5:26803215:26805384:-1
Orangutan	chromosome:PPYG2:17:35636585:35638674:-1
Gibbon	chromosome:Nleu_3.0:14:7107226:7110428:1
Vervet-AGM	chromosome:ChlSab1.1:16:36817797:36819808:-1
Crab-eating macaque	chromosome:Macaca_fascicularis_5.0:16:42070100:42073792:1
Macaque	chromosome:Mmul_8.0.1:16:33084189:33086199:-1
Olive baboon	chromosome:Panu_3.0:16:37660452:37662462:1
Marmoset	contig:ASM275486v1:NTIC01034389.1:103269761:103271803:-1
Mouse Lemur	chromosome:Mmur_3.0:16:19873365:19875382:-1
Prairie vole	chromosome:MicOch1.0:7:65526589:65528566:-1
Ryukyu mouse	chromosome:CAROLI_EIJ_v1.1:11:84562720:84564837:-1
Mouse	chromosome:GRCm38:11:89301643:89303693:-1
Algerian mouse	chromosome:SPRET_EiJ_v1:11:90011827:90013820:-1
Shrew mouse	chromosome:PAHARI_EIJ_v1.1:14:56973050:56975047:-1
Rat	chromosome:Rnor_6.0:10:76812760:76814845:-1
Rabbit	chromosome:OryCun2.0:19:31415564:31418130:-1
Cow	chromosome:UMD3.1:19:7611906:7612095:1
Goat	chromosome:ARS1:19:6994850:6996672:1
Sheep	chromosome:Oar_v3.1:11:6945324:6947041:1
Pig	chromosome:Sscrofa11.1:12:32889539:32891386:1
Horse	chromosome:EquCab2:11:31050972:31053118:1
Dog	chromosome:CanFam3.1:9:31453386:31455737:1
Cat	chromosome:Felis_catus_9.0:E1:32063911:32066200:-1
